# Advancing species-level vegetation mapping of alpine peat bogs using UAV imagery, machine learning and texture features

**DOI:** 10.3389/fpls.2026.1841696

**Published:** 2026-06-29

**Authors:** Adam Kulich, Lucie Červená, Jakub Lysák, Lucie Kupková

**Affiliations:** Department of Applied Geoinformatics and Cartography, Faculty of Science, Charles University, Prague, Czechia

**Keywords:** alpine peat bogs, machine learning, species composition, species-level vegetation mapping, UAV multispectral imagery

## Abstract

Alpine peat bogs are valuable, highly heterogeneous wetland ecosystems with specialized and often rare species, where spatial variation in species composition reflects fine-scale hydrological and ecological gradients. They play a key role as natural water and carbon reservoirs and contribute to landscape resilience under climate change, yet are highly vulnerable to drainage, droughts, and chemical changes, leading to vegetation shifts and biodiversity loss. Reliable species-level vegetation monitoring is therefore essential for understanding these dynamics and supporting conservation and management. This study evaluates the potential of UAV-based multispectral imagery combined with machine learning and texture features for species-level vegetation mapping in alpine peat bogs. Two representative peat bogs in the Krkonoše Mountains - the Úpské and Hraniční Meadow Peat Bogs, located within a Ramsar-listed wetland - were surveyed, with high-precision ground-truth data collected for 14 vegetation classes. Four classifiers (Random Forest, Gradient Boosting, XGBoost, and Multi-Layer Perceptron) were compared, and texture features derived from GLCM, GLDM, and GLSZM matrices were systematically assessed. XGBoost achieved the highest accuracy (weighted and macro F1-scores up to 94% and 79% respectively), while ensemble approaches enabled spatial consistency mapping. By jointly evaluating classifiers, texture features, and multi-temporal data, the study provides a comprehensive assessment of factors influencing species-level classification accuracy. Integrating texture features and multi-temporal UAV data significantly improves classification performance and enables detailed mapping of species composition, which serves as an indicator of ecosystem dynamics and associated environmental conditions. The proposed approach supports high-resolution, long-term monitoring of alpine peatland vegetation and improves the detection of fine-scale changes, contributing to more effective conservation and management of these vulnerable ecosystems.

## 1 Introduction

Alpine peat bogs are among the most sensitive ecosystems, characterized by strong spatial heterogeneity and the occurrence of specialized and often rare species, where small changes in species composition can be interpreted as indicators of shifts in hydrological and ecological conditions (Davidson, 2014; Junk et al., 2013; Zedler and Kercher, 2005). These ecosystems play a crucial role as natural water and carbon reservoirs and contribute to landscape resilience under climate change (Fraser and Keddy, 2005; Chmura et al., 2003; Mitsch et al., 2014). However, they are highly vulnerable to drainage, groundwater depletion, droughts, and changes in chemical regimes, which may lead to vegetation shifts, the loss of rare species and biodiversity (Davidson, 2014; Zedler and Kercher, 2005; Mitsch et al., 2014; Chambers et al., 2001; Vacek et al., 1998). Reliable species-level vegetation monitoring is therefore essential for understanding these processes and supporting conservation and management.

Traditional vegetation assessment based on detailed field surveys provides accurate species-level information (Huylenbroeck et al., 2020), but is time-consuming, labor-intensive, and costly, limiting spatial coverage and temporal frequency (Reinke, 2006; Rhodes et al., 2015; Rooney et al., 2012; Ko et al., 2017). As a result, monitoring species composition and its temporal changes across larger areas remains challenging. Remote sensing (RS), including multispectral and hyperspectral data from satellite, aerial, and increasingly Unmanned Aerial Vehicles (UAV), enables repeated observations over large areas and represents an effective alternative for long-term vegetation monitoring.

Satellite and aerial imagery remain the most widely used approaches (Huylenbroeck et al., 2020), with a long tradition (e.g., Driscoll and Spencer, 1972), typically applied at community or ecosystem scales (Kaplan and Avdan, 2017; Wei et al., 2022; Mishra et al., 2021), but also for species-level mapping (Behera et al., 2021; Chen et al., 2024; Kupková et al., 2023) and invasive species detection (Skowronek et al., 2017; Elmer et al., 2021; Sabat-Tomala et al., 2022; Kupková et al., 2025). UAVs are increasingly used where very high spatial resolution is required, such as detailed species-level mapping or individual plant detection (Windle et al., 2023; Kaplan and Avdan, 2018; Abeysinghe et al., 2019; Kupková et al., 2023). Their flexibility and relatively low cost (Jeziorska, 2019; Pricope et al., 2024; Huylenbroeck et al., 2020) make them a suitable compromise between field surveys and satellite or aerial observations, particularly in inaccessible or sensitive small-scale environments (Alvarez-Vanhard et al., 2020; Jeziorska, 2019; DiGiacomo et al., 2022; Anderson et al., 2023).

In the context of wetland vegetation mapping specifically, satellite-based approaches have demonstrated considerable utility at community and ecosystem scales (Kaplan and Avdan, 2017; Wei et al., 2022; Mishra et al., 2021). Multi-temporal Sentinel-2 time series have proven effective for capturing the phenological dynamics of aquatic and semi-aquatic plant assemblages (Piaser and Villa, 2023), yet the spatial resolution of freely available imagery fundamentally constrains species-level discrimination in highly heterogeneous wetlands where community patches occur at sub-metre scales (Huylenbroeck et al., 2020). Aircraft-based hyperspectral data can overcome this resolution limitation (Behera et al., 2021; Marcinkowska-Ochtyra et al., 2018), and their fusion with polarimetric SAR and LiDAR has enabled high-precision classification in wetland types such as mangroves, coastal marshes, and karst wetlands (Yao et al., 2025; Guo et al., 2023; Fu et al., 2023). However, the associated acquisition costs and logistical requirements can be prohibitive for repeated, long-term aircraft-based monitoring in remote mountain environments (Huylenbroeck et al., 2020; Jeziorska, 2019; Rhodes et al., 2015). UAV platforms offer a practical resolution to these constraints, combining high spatial detail with flexible, low-cost deployment (Alvarez-Vanhard et al., 2020; DiGiacomo et al., 2022; Jeziorska, 2019).

Despite these advantages, UAV technology remains underutilized in protected areas, often due to limited collaboration between RS experts and environmental management practitioners (Walker et al., 2022; Rossi and Wiesmann, 2024; Seier et al., 2021). UAVs have been used in only about 10% of RS-based wetland studies, although this proportion is increasing (Huylenbroeck et al., 2020). Existing applications focus on mapping plant communities (Pricope et al., 2023; Alvarez-Vanhard et al., 2020; Bhatnagar et al., 2021; Liu and Abd-Elrahman, 2018a), species (Cao et al., 2018; Pande-Chhetri et al., 2017; Gonzalez-Perez et al., 2022), invasive species (Evans et al., 2024; Anderson et al., 2023; Wan et al., 2014; Jochems et al., 2021), wetland extent (Nuradili et al., 2024; Qiu et al., 2025), or water levels (Arroyo-Mora et al., 2023; Góraj et al., 2019; Perivolioti et al., 2022).

A key step in vegetation mapping is RS-based classification, whose performance depends on multiple factors, including data acquisition, input features, training data quality, classifier choice, and training strategy (Sabat-Tomala et al., 2022; Zagajewski et al., 2021; Maxwell et al., 2018; Jochems et al., 2021). In addition to spectral information, classification can incorporate derived indices (Kaplan and Avdan, 2018; Abeysinghe et al., 2019), canopy height (Abeysinghe et al., 2019; Jochems et al., 2021), and texture features (Michez et al., 2016; Mohammadpour et al., 2022; Kupková et al., 2023). Texture features derived from GLCM (Haralick et al., 1973), GLDM (Sun and Wee, 1983), and GLSZM (Thibault et al., 2009) capture spatial context, but their combined effectiveness for mapping low-growing peatland vegetation remains insufficiently explored, despite some recent studies (Erdem and Bayrak, 2023).

Classifier selection is equally important. Traditional statistical approaches such as Maximum Likelihood (Richards and Jia, 2006; Bolstad and Lillesand, 1991; Sisodia et al., 2014) are generally outperformed by modern machine learning methods capable of modelling complex, non-linear relationships (Lu and Weng, 2007; Maxwell et al., 2018). Widely used approaches include Random Forest (Breiman, 2001) and Support Vector Machines (Cortes and Vapnik, 1995), both demonstrating high performance in RS applications (Belgiu and Drăguţ, 2016; Pal and Mather, 2005; Mountrakis et al., 2011; Dabija et al., 2021). In wetland vegetation mapping specifically, Random Forest has been widely adopted, demonstrating strong performance in species-level classification of coastal marshes (Windle et al., 2023), mapping of invasive wetland vegetation (Jochems et al., 2021; Abeysinghe et al., 2019), and species-level peatland monitoring from UAV imagery (Hamedianfar et al., 2025; Kupková et al., 2023). Support Vector Machines have similarly been applied for object-based classification of wetland plant communities (Pande-Chhetri et al., 2017; Liu and Abd-Elrahman, 2018a; Du et al., 2021) and wetland invasive species detection (Abeysinghe et al., 2019), showing competitive accuracy particularly when training data are limited. Comparative studies across wetland environments have, however, consistently shown that no single classifier universally outperforms all others, and that suitability varies with data type (Maxwell et al., 2018; Khatami et al., 2016), spatial resolution (Huylenbroeck et al., 2020; Kaplan and Avdan, 2017), class complexity (Zagajewski et al., 2021; Jochems et al., 2021), and training sample availability (Ma et al., 2019; Hamedianfar et al., 2025).

More recently, advanced methods such as XGBoost (Chen and Guestrin, 2016; Zhang et al., 2019; Zhen et al., 2024; Hamedianfar et al., 2025) and Convolutional Neural Networks have been increasingly applied, often outperforming traditional approaches (Zhang et al., 2019; Yoon and Jung, 2024; Zhen et al., 2024; Sothe et al., 2020; Yang et al., 2020). XGBoost in particular has demonstrated state-of-the-art performance across a range of wetland and vegetation mapping contexts, including mangrove species classification (Zhen et al., 2024) and peatland vegetation mapping from UAS-derived data, where it outperformed both RF and SVM under conditions of limited training data and high class complexity (Hamedianfar et al., 2025). Convolutional Neural Networks have also been explored in wetland contexts, showing advantages in capturing spatial patterns in heterogeneous environments (Liu et al., 2018b; Gonzalez-Perez et al., 2022), though their data requirements often pose challenges in ecologically sensitive areas where ground-truth collection is constrained (Ma et al., 2019; Li et al., 2017). While most methods operate at the pixel level, spatial context can be further incorporated using texture features, object-based approaches (Blaschke, 2010; Hay and Castilla, 2008; Duro et al., 2012), or convolutional architectures.

This study focuses on species-level vegetation classification in alpine peat bogs in the Krkonoše Mountains using high-resolution UAV imagery, machine-learning algorithms, texture-based features derived from gray-level matrices, and ground-truth botanical data for training and validation. Peatland vegetation in mountain environments is structurally complex and spatially heterogeneous, which makes species-level discrimination particularly challenging. Although remote sensing and artificial intelligence methods have increasingly advanced vegetation mapping across a range of ecosystems, peat bogs remain comparatively less studied at the species level.

Within Europe, examples of species-level mapping of peatland vegetation include work at the Auchencorth Moss site in Scotland (Simpson et al., 2024), where a Maximum Likelihood classifier yielded an overall accuracy of 69%. In the Krkonoše National Park, remote sensing has previously been applied to alpine habitats above the treeline using satellite imagery (Kupková et al., 2017; Kupková et al., 2023), aerial photographs (Kupková et al., 2017; Potůčková et al., 2021; Marcinkowska-Ochtyra et al., 2017; Marcinkowska-Ochtyra et al., 2014; Marcinkowska-Ochtyra et al., 2018), and UAV-based observations (Kupková et al., 2023). However, these studies primarily targeted broader vegetation units and landscape-level patterns rather than peat-bog species.

Species-level mapping in the Krkonoše Mountains has so far been conducted only within a previous UAV-based tundra vegetation monitoring project (2019–2023), which covered a 1-ha plot of the Úpské Peat Bog using multispectral UAV imagery and achieved an overall accuracy of 85.8% (Kupková et al., 2020). Overall, species-level mapping of alpine peatlands remains limited, and no study has systematically evaluated multiple classifiers in combination with texture-based features.

Addressing these limitations, the main objective of this study was to advance species-level mapping of alpine peat bog vegetation by evaluating how different machine learning algorithms, in combination with texture-based features, improve the representation of plant species in heterogeneous environments. The study focuses on two representative peat bogs within the Krkonoše/Karkonosze Subalpine Peatbogs Ramsar Site and aims to develop a robust and transferable workflow for detailed vegetation mapping in ecologically sensitive mountain ecosystems.

Specifically, we aimed to:

1. compare the classification performance of several per-pixel machine learning algorithms, including Random Forest, Gradient Boosting Classifier, XGBoost, and Multi-Layer Perceptrons, to identify the most suitable method for species-level mapping of alpine peat bog vegetation;
2. evaluate the contribution of texture-based features derived from GLCM, GLDM, and GLSZM matrices by testing their individual and combined effects on classification accuracy;
3. develop a methodological framework that supports reliable, long-term monitoring of alpine peat bog vegetation, with a focus on species composition as an indicator of ecosystem dynamics.

As one of the main outcomes, the study delivers a detailed species-level vegetation map for two alpine peat bogs in the Krkonoše Mountains, produced using the best-performing machine learning classification approach in combination with the most effective texture-based features identified during model evaluation. This map serves as a practical demonstration of the proposed approach, while the elaborated methodological framework provides a transferable basis for high-resolution vegetation biodiversity monitoring and supports management and conservation efforts in alpine peatland ecosystems.

## 2 Materials and methods

### 2.1 Study area

Alpine peat bogs represent one of the key conservation priorities within the Krkonoše National Park (KRNAP) in northern Czechia. Characteristic flora and fauna, the average annual temperature (close to 0.2 °C) and precipitation (~1800 mm) are comparable to conditions found in arctic peat bogs of Scandinavia (Jobda et al., 2019; Jeník and Soukupová, 1992). These ecosystems are remnants of the last Ice Age and host numerous relict species (Jeník, 1961; Dolný, 2013; Hadac & Vána, 1967). In addition to being protected as a national park, the Krkonoše Mountains are designated as a UNESCO Transboundary Biosphere Reserve and form part of the EU-wide Natura 2000 conservation network. The peat bogs investigated in this study are further recognized as wetlands of international importance under the Ramsar Convention (Jobda et al., 2019). These fragile ecosystems, located above the tree line, are subject to multiple anthropogenic threats, including climate change, air pollution, and historical drainage activities that have lowered groundwater levels (Volf et al., 2019). In recent years, conservation initiatives have increasingly focused on the revitalization and long-term preservation of peat bogs.

**Figure 1:**
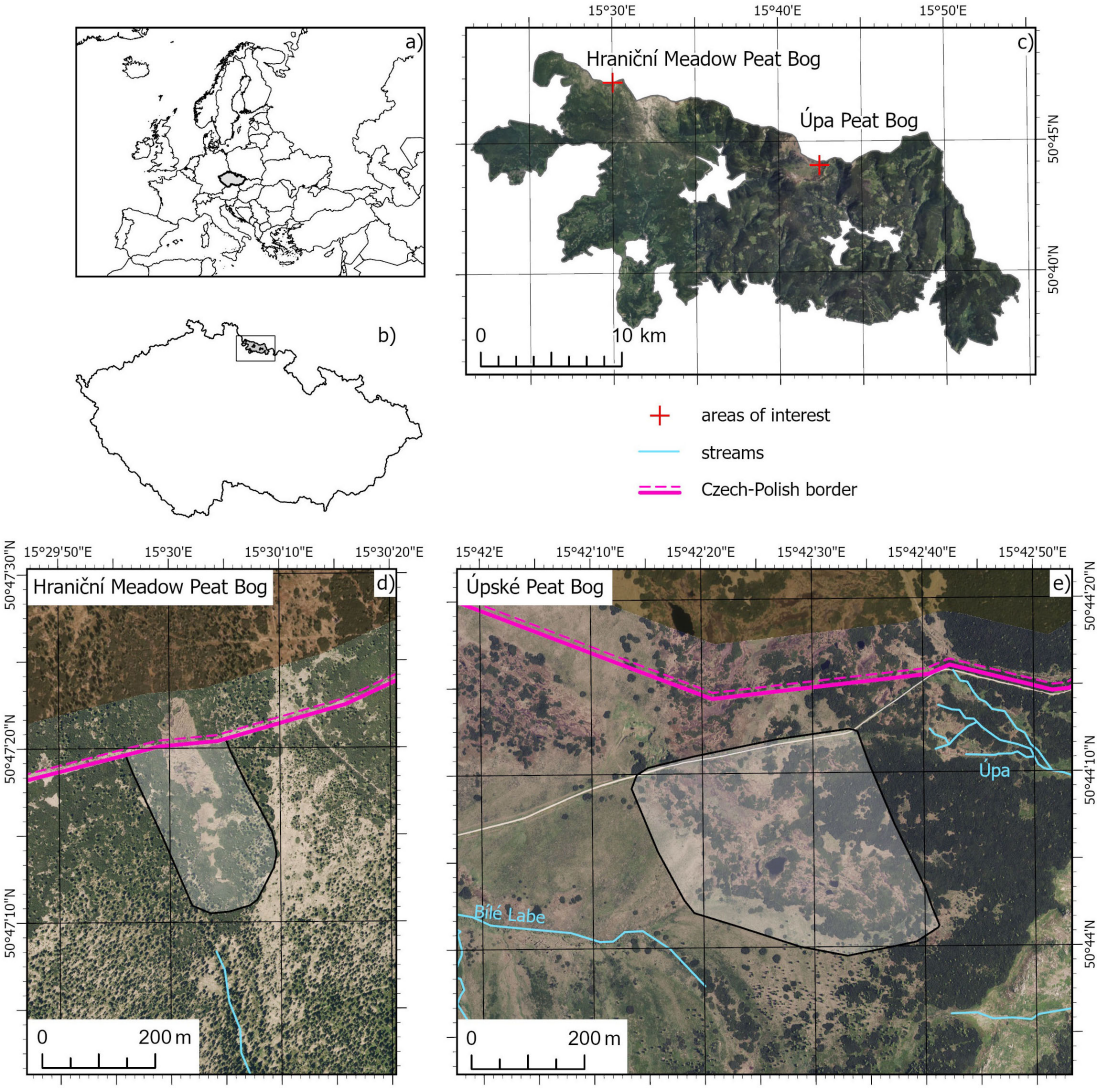
Location of the study areas: **(a)** Czech Republic in Europe, **(b)** Krkonoše Mountains in the Czech Republic, **(c)** Study areas in the Krkonoše National Park, **(d)** Hraniční Meadow Peat Bog and **(e)** Úpské Peat Bog in the Czech Republic (ČÚZK, 2024; GUGiK, 2025).

This study focuses on two peat bogs within the KRNAP (**Figure 1**): the Hraniční Meadow Peat Bog (*Hraniční louka*) and the Úpské Peat Bog (*Úpské rašeliniště*). The Hraniční Meadow Peat Bog is situated in the western part of the Krkonoše Mountains at an altitude of approximately 1240 m a.s.l., while the Úpské Peat Bog, the largest peat bog in the range, lies in its eastern part at over 1420 m a.s.l. Both sites share a characteristic dominant species composition typical of subalpine mires, including *Trichophorum cespitosum*, *Carex* and *Sphagnum* species, with *Pinus mugo* and *Picea abies* in the drier areas and the bog margins (Jeník and Soukupová, 1992; Hadac & Vána, 1967). The two sites were selected to represent the main altitudinal and structural gradient of peat bog types occurring in the Krkonoše Mountains, with Úpské Peat Bog representing a higher-altitude ombrotrophic bog and Hraniční Meadow Peat Bog representing a lower-altitude transitional mire type. Together they account for two of the three peat bogs constituting the Czech part of the Krkonoše/Karkonosze Subalpine Peatbogs Ramsar Site, underscoring their exceptional conservation value and representativeness within the broader protected area. Their species composition, vegetation structure, and hydrological conditions are broadly representative of subalpine peat bogs occurring across Central European mountain ranges such as the Tatras, Šumava, and Erzgebirge, supporting the potential transferability of the methodological framework to comparable ecosystems in the region (Jeník and Soukupová, 1992; Jobda et al., 2019). Both sites contribute critically to the hydrological function of the Krkonoše Mountains, serving as natural water reservoirs that regulate streamflow in the headwater catchments of the Úpa and Bílé Labe rivers, while also representing significant long-term carbon stores and refugia for rare and endangered species of both flora and fauna (Jobda et al., 2019; Fraser and Keddy, 2005; Chmura et al., 2003).

The Úpské Peat Bog is particularly notable for its glacial relict species including *Rubus chamaemorus*, *Pedicularis sudetica*, and *Carex lindbergii*, representing some of the most botanically significant elements of the Krkonoše flora (Jeník, 1961; Dolný, 2013), while the Hraniční Meadow Peat Bog represents a lower-altitude, structurally distinct peat bog type with rare stands of *Drosera rotundifolia.* While these relict species highlight the exceptional conservation value of the site, they occur sparsely and at low spatial densities, precluding their inclusion as discrete classification targets in this study. Although both sites extend across the Czech–Polish border, only the Czech parts were included in this study, covering 9 ha at Úpské and 2.5 ha at Hraniční Meadow Peat Bog.

### 2.2 Data collection and classification legend

Two types of datasets were acquired at each study site during the summer of 2024: UAV image data (**Table 1**) and botanical ground-truth data (**Tables 2**, **3**). The first dataset comprised UAV multispectral imagery collected using the DJI Mavic 3M and the DJI Phantom 4 Multispectral for the Úpské Peat Bog and the DJI Mavic 3M for Hraniční Meadow Peat Bog. All image data were georeferenced in real time using Real-Time Kinematic (RTK) positioning to ensure high spatial accuracy.

**Table 1:** Information about the image data acquired.

Area	Date	Overlap (%)	Spatial resolution	UAV type	Weather conditions	Mean horizontal error
Úpské Peat Bog	10.7.2024	85, 70	3.5 cm	DJI Mavic 3M	Mostly sunny	3.08 cm
Úpské Peat Bog	7.8.2024	80, 80	3.5 cm	DJI Phantom 4 MS	Mostly sunny	4.95 cm
Hraniční Meadow Peat Bog	9.8.2024	85, 70	3 cm	DJI Mavic 3M	Sunny	3.16 cm

Horizontal accuracy was measured on control points (see Section 2.3).

**Table 2:** Classification legend for the Úpské Peat Bog.

Class	Number of measurements	Number of all ground truth pixels	Proportion of pixels (*) (%)	Area proportion (**) (%)
*Calluna vulgaris* (Common heather)	78	6110	2, 40	5.50
*Carex limosa* (Mud sedge)	66	15407	6, 06	7.03
*Carex rostrata* (Beaked sedge)	45	15753	6, 19	3.80
*Eriophorum angustifolium *(Common cottongrass)	9	3052	1, 20	0.09
*Juncus spp.* (Rushes)	6	436	0, 17	0.30
*Molinia caerulea* (Purple moor-grass)	26	9470	3, 72	3.90
*Nardus stricta* (Mat-grass)	38	16990	6, 68	22.80
*Picea abies* (Norway spruce)	14	8976	3, 53	0.20
*Pinus mugo* (Mountain pine)	185	118611	46, 63	37.600
*Sphagnum spp.* (Peat moss)	8	731	0, 29	0.05
*Trichophorum cespitosum *(Tufted bullrush)	156	47047	18, 50	15.60
*Vaccinium myrtillus *(European blueberry)	6	487	0, 19	0.007
*Vaccinium uliginosum* (Bog bilberry)	21	1699	0, 67	1.20
*Water and mud*	34	9572	3, 76	1.8

*Out of all pixels before stratified sampling; **according to the initial classification).

**Table 3:** Classification legend for the Hraniční Meadow Peat Bog.

Class	Number of measurements	Number of all ground truth pixels	Proportion of pixels (*) (%)	Area proportion (**) (%)
*Carex limosa *(Mud sedge)	10	1553	1, 93	5.60
*Carex rostrata *(Beaked sedge)	7	2025	2, 51	6.50
*Empetrum spp. *(Black crowberry)	5	305	0, 38	0.45
*Molinia caerulea *(Purple moor-grass)	5	2129	2, 64	6.00
*Picea abies *(Norway spruce)	34	27995	34, 72	12.20
*Pinus mugo *(Mountain pine)	35	30543	37, 88	50.60
*Sphagnum spp.* (Peat moss)	9	2256	2, 80	3.60
*Trichophorum cespitosum *(Tufted bullrush)	18	6423	7, 97	9.90
*Vaccinium myrtillus *(European blueberry)	4	593	0, 74	1.50
*Vaccinium uliginosum *(Bog bilberry)	5	638	0, 79	0.60
Unidentified moss	4	209	0, 26	1.40
Dry vegetation	5	1100	1, 36	0.30
Water and mud	12	4861	6, 03	1.60

*Out of all pixels before stratified sampling; **according to the initial classification.

The DJI Phantom 4 Multispectral is equipped with a camera that captures five discrete spectral bands: Blue (450 ± 16 nm), Green (560 ± 16 nm), Red (650 ± 16 nm), Red Edge (730 ± 16 nm), and Near-Infrared (NIR, 840 ± 26 nm) (DJI, 2019). The DJI Mavic 3M camera provides four spectral bands: Green (560 ± 16 nm), Red (650 ± 16 nm), Red Edge (730 ± 16 nm), and NIR (860 ± 26 nm) (DJI, 2022). For the Hraniční Meadow Peat Bog, only one set of image data was captured, while for the Úpské Peat Bog, data were acquired on two dates. Detailed flight specifications are summarized in **Table 1**.

In addition to the image data, ground-truth data for individual plant species were collected in the field with the support of botanists from KRNAP. High-precision geolocation was ensured using Trimble R8 and R10 GNSS receivers, achieving horizontal accuracy between 5 and 20 mm. The data points were labelled with the assigned species class and the diameter of the area in which the species was dominant with 100% cover. For some visually distinct species (e.g., *Pinus mugo*, *Picea abies*), the reference data were manually derived from high-resolution orthomosaics. Ground-truth data for both sites were then converted into circle-shaped vector polygon layers, using the diameter attributes. The complete classification legends, including the number of measurements, total ground-truth pixels, and class proportions, are provided in **Tables 2**, **3** for the Úpské Peat Bog and the Hraniční Meadow Peat Bog, respectively. A comparison of area proportions between the initial and most accurate classifications is provided in **Supplementary Appendix C**.

### 2.3 Data preprocessing

The UAV multispectral images were pre-processed in Pix4D. Image alignment, radiometric correction (using image metadata and irradiance sensor data), and photogrammetric processing were performed to generate point clouds, digital surface models (DSM), and seamless orthomosaics for each date and site. Ground control points were used to assess and improve spatial accuracy. A canopy height model (CHM) was derived by subtracting the digital terrain model (DMR5G; Český Úřad Zeměměřický a Katastrální (ČÚZK), 2016) from the DSM. The dataset from 7 August yielded consistently lower performance in initial testing and was therefore excluded from standalone classification to reduce computational demands but was retained as an additional temporal layer in the multi-temporal feature stack. It should be noted that, as the two acquisitions at Úpské Peat Bog were collected with different sensors, the multi-temporal feature stack assumes cross-sensor spectral comparability that radiometric correction alone cannot fully guarantee.

Texture features were calculated for each spectral band and the CHM using three matrix types: GLCM, GLDM, and GLSZM. GLCM (Haralick et al., 1973) describes the frequency of co-occurring pixel values at a given spatial offset. GLDM (Sun and Wee, 1983) quantifies local pixel dependency based on intensity similarity within a kernel. GLSZM (Thibault et al., 2009) captures the size distribution of connected regions with identical gray levels, independent of direction. Together, these approaches represent complementary spatial characteristics of the data. All texture calculations depend on kernel size, which defines the neighborhood used for analysis.

Based on initial testing (kernel sizes 3–13), a 7×7 kernel was selected as a compromise between contextual information and classification performance. For each input layer, comprising spectral bands and the CHM, nine texture features were derived following Hall-Beyer (2017): mean, standard deviation, correlation, contrast, dissimilarity, homogeneity, energy, maximum, and entropy. Applied across all input layers, this yielded 45 texture features per matrix type for each site (Mavic 3M: 4 spectral bands + CHM = 5 input layers × 9 metrics). Across all three matrix types combined, this resulted in 135 texture features per site, which together with the original spectral bands and CHM formed the complete feature set for classification.

Ground-truth data were split into spatially representative training and test sets using stratified sampling. For the Úpské Peat Bog dataset, 20% of observations were allocated to an independent test set, which was sufficient given the sample size (Géron, 2022; Kuhn and Johnson, 2013). For the smaller Hraniční Meadow Peat Bog dataset, this proportion was increased to 33% to improve the reliability of performance estimates and representation of rare classes (Goodfellow et al., 2016). The remaining data were divided into k-fold cross-validation subsets (five folds for Úpské Peat Bog, three for Hraniční Meadow Peat Bog) for model selection and hyperparameter tuning.

To reduce spatial autocorrelation, folds were created spatially following best practices in remote sensing (Roberts et al., 2017). Within each class, polygons were first ordered by iterative spatial selection: at each step, the polygon maximizing the minimum distance to all already-selected polygons was chosen next, producing a sequence ranked by spatial separation. Folds were then assigned sequentially based on this ranked order using a cyclic assignment scheme (rank modulo number of folds), such that consecutive ranks were assigned to different folds. This approach aims to promote spatial diversity within each fold by distributing polygons across the range of pairwise separations, rather than concentrating them in spatially proximate clusters. Individual polygons were never split between datasets. All folds were visually inspected for spatial consistency, though no explicit distance threshold was imposed and the degree of residual spatial autocorrelation between folds was not formally quantified. Class separability was assessed using the Jeffries–Matusita index (Richards & Jia, 1999) to confirm the feasibility of classification 0(Appendix E).

Initial classifications using Random Forest were performed to estimate class area proportions, which were used to guide stratified sampling of training, validation, and test data. This approach was adopted also to ensure sufficient representation of rare classes that would be severely undersampled by purely random sampling. The initial classification influenced only the sampling weights and not the ground-truth labels, which were derived exclusively from field-collected botanical data, and the independent test set was withheld prior to any model training, ensuring the final accuracy assessment remained unaffected by this procedure. Pixels were sampled from ground-truth polygons according to class area in the preliminary map. For rare classes (<1% area), a minimum representation of 1% was enforced; if not achievable, all available pixels were used. This ensured sufficient representation of minority classes. A complete list of vegetation classes is provided in **Tables 2**, **3**. Before model training, all features were standardized (zero mean, unit variance) to ensure stable and comparable model performance.

### 2.4 Classifiers

Four widely used machine learning classifiers were applied. Random Forest (RF; Breiman, 2001) is an ensemble method combining multiple decision trees built on bootstrap samples and random feature subsets to reduce variance and overfitting. Gradient Boosting Classifier (GBC; Friedman, 2001) builds trees sequentially, each fitted to the residuals of the previous model, improving performance through iterative loss minimization. Extreme Gradient Boosting (XGBoost/XGB; Chen and Guestrin, 2016) is an optimized gradient boosting implementation incorporating regularization and efficient handling of sparse data, often enhancing accuracy and scalability. Multi-Layer Perceptron (MLP; Rumelhart et al., 1986) is a feedforward neural network capable of modelling complex nonlinear relationships using backpropagation.

These classifiers were selected to represent a complementary spectrum of machine learning approaches: RF and GBC as well-established ensemble methods with consistently strong performance in vegetation and wetland classification (Belgiu and Drăguţ, 2016; Maxwell et al., 2018; Hamedianfar et al., 2025), XGBoost as an optimized boosting implementation increasingly recognized as state-of-the-art for species-level mapping tasks (Zhang et al., 2019; Zhen et al., 2024; Hamedianfar et al., 2025), and MLP as a representative neural network approach capable of capturing complex non-linear feature interactions (Goodfellow et al., 2016). Additional classifiers including Support Vector Machines, Convolutional Neural Networks and Object-Based classifiers using Support Vector Machines and Random Forest were also tested during preliminary evaluation but were ultimately excluded due to prohibitive computational demands and failure to learn rare classes, as discussed in Section 4. All selected classifiers were further optimized through grid search hyperparameter tuning with k-fold cross-validation, as described in Section 2.6. To further assess generalization performance, ensemble approaches were also tested (Dietterich, 2000), including a 10-MLP ensemble and a stacked ensemble combining all applied classifiers.

### 2.5 Texture testing

Texture features were evaluated using k-fold cross-validation (three folds for the Hraniční Meadow Peat Bog, five for the Úpské Peat Bog). Based on initial testing and established recommendations (Breiman, 2001; Friedman, 2001; Hastie et al., 2009; Chen and Guestrin, 2016; Bishop, 1995; Goodfellow et al., 2016), RF was implemented with 500 trees and √p feature sampling. XGB and GBC used 200 trees with a learning rate of 0.1. The MLP consisted of a single hidden layer with 100 neurons and the Adam optimizer (Kingma and Ba, 2014).

Testing datasets were generated by incrementally adding texture features from individual matrices and their combinations to the multispectral orthomosaic and CHM. Models were trained and evaluated across these configurations, and average cross-validation F1-scores were used to identify the most informative texture feature sets.

### 2.6 Model training and fine tuning

Final vegetation maps were produced using multiple trained and fine-tuned models. Model performance was primarily evaluated using weighted F1-score, complemented by overall accuracy and class-level precision and recall. Hyperparameter tuning was performed via grid search with k-fold cross-validation to prevent overfitting, and final performance was assessed on independent test sets. The full set of tested hyperparameters is provided in **Supplementary Appendix A**.

Fine tuning was conducted for RF, GBC, XGB, and MLP, and the best-performing model of each type was used for mapping. For the Úpské Peat Bog, tuning was performed on both a single-date dataset (10 July; MS) and a multi-temporal dataset (MT) created by stacking of the original bands from 7 August on top of the single-date dataset.

### 2.7 Feature interpretability analysis

To assess feature contributions and provide interpretability beyond classification accuracy, we applied two complementary *post-hoc* explainability methods to the best-performing XGBoost models at each site. SHAP (SHapley Additive exPlanations) values were computed using TreeExplainer (Lundberg and Lee, 2017) on a stratified subsample of 3, 500 test pixels per site, decomposing model predictions into per-feature contributions. LIME (Local Interpretable Model-agnostic Explanations) (Ribeiro et al., 2016) was also applied to the same stratified test instances. Feature contributions were aggregated by feature category (spectral, GLCM, GLSZM, GLDM) to quantify the relative importance of each data type. Per-class rankings were also derived to identify class-specific discriminative feature signatures.

### 2.8 Ensembling

**Figure 2:**
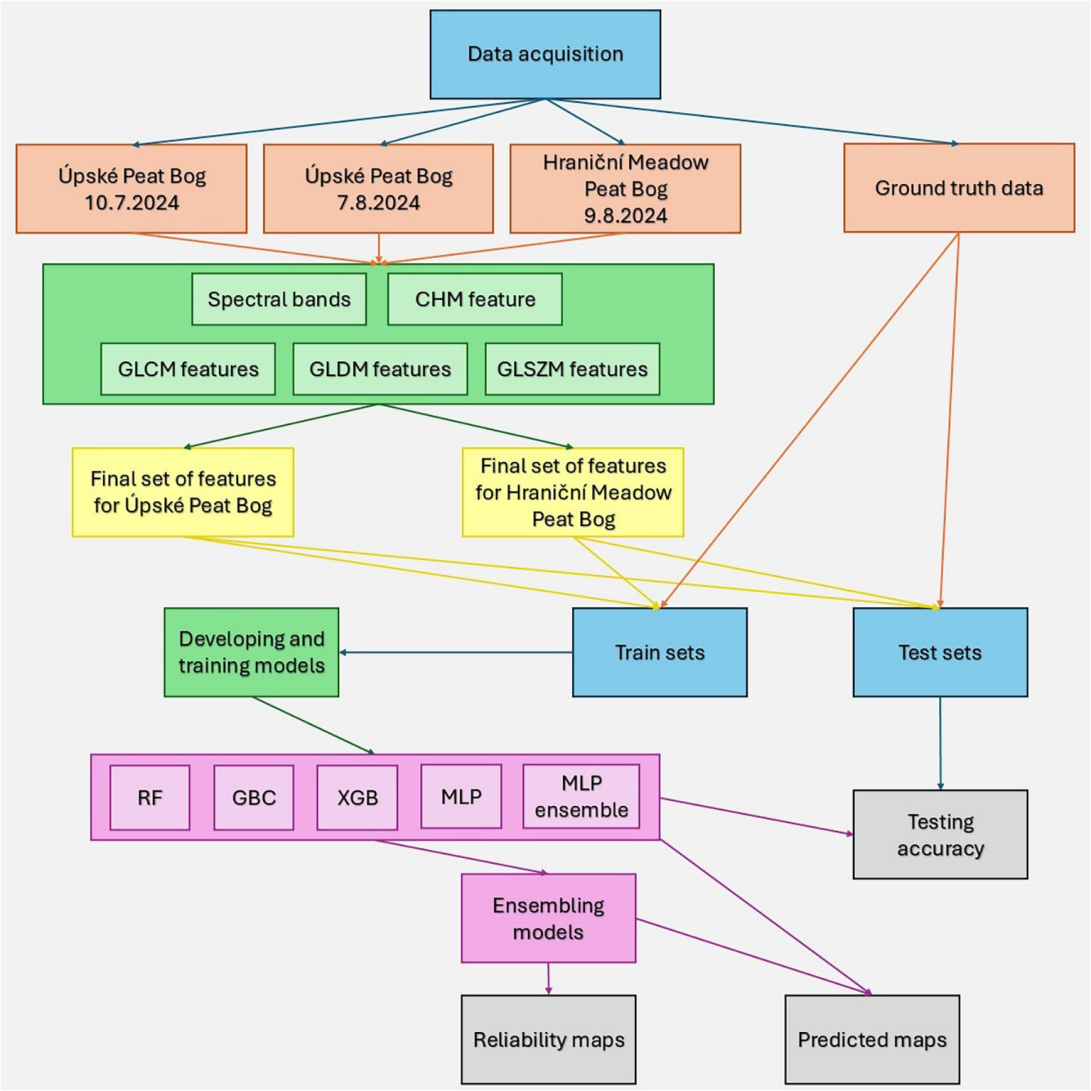
Data processing chart.

As a final step, a majority-voting ensemble was constructed using all individual classifiers that achieved a weighted F1-score above 90%. The predictive accuracy of this ensemble was then compared with that of the best-performing single model. Additionally, the ensemble was employed to assess spatial classification agreement, quantified as the proportion of classifiers concurring on the predicted label for each pixel. The resulting agreement map delineates regions of high and low model consensus, providing a spatially explicit measure of the consistency of species predictions across the study area. A summary of the whole analysis is provided in **Figure 2**.

## 3 Results

**Figure 3:**
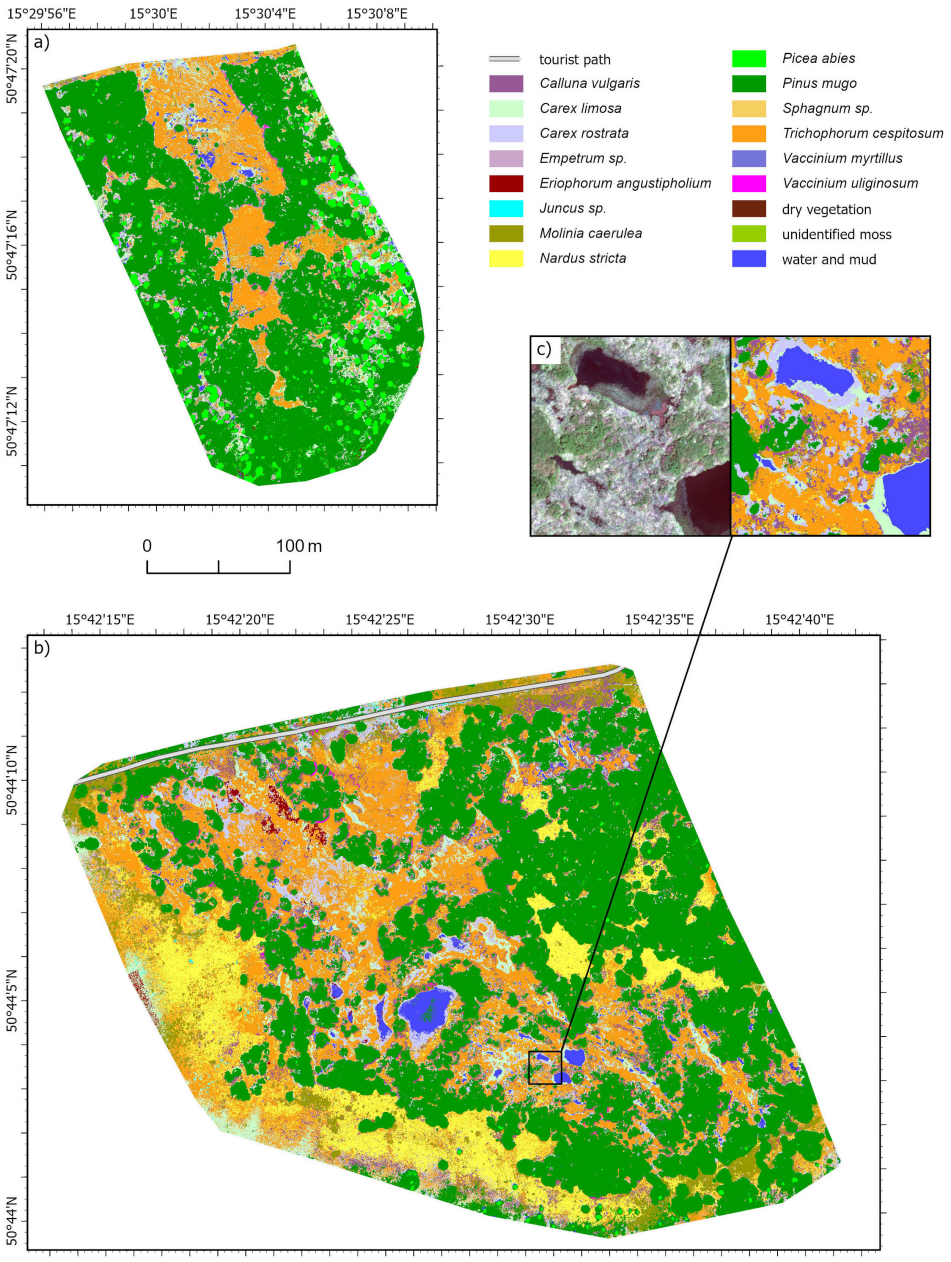
Best classification results: XGBoost classification of the Hraniční Meadow Peat Bog **(a)**, ensemble classification of the Úpské Peat Bog **(b)** and detailed comparison with the UAV orthomosaic **(c)**. **Supplementary Appendix D** contains maps for both sites in better resolution.

The Results section is structured to follow the methodological workflow. First (Section 3.1), we assess the effect of texture feature sets on classifier performance. Then (Section 3.2), we report the outcomes of model fine-tuning and classification accuracy. Finally (Section 3.3), we evaluate ensemble models and consistency mapping. Special attention is paid to the comparison between single- and multi-temporal datasets and to differences in classifier performance across the two study sites. The final vegetation maps are presented in **Figure 3**, and summary statistics are provided in **Tables 4**–**7**.

**Table 4:** Best model accuracies and weighted/macro F1-scores respectively (P.B., Peat Bog).

Dataset	RF		GBC		XGB		MLP		MLP ens.	
	OA	F1	OA	F1	OA	F1	OA	F1	OA	F1
Úpské P. B. (MS)	88.5	88.5/71.0	88.5	88.4/71.4	90.9	91.0/76.7	87.2	86.9/68.8	89.1	88.9/70.8
Úpské P. B. (MT)	92.5	92.2/74.8	92.3	92.0/74.5	94.2	93.9/78.6	91.5	91.5/71.2	93.7	93.6/76.5
Hraniční Meadow P. B.	87.7	86.5/58.5	87.5	87.2/55.1	90.2	89.3/61.3	83.0	81.5/55.6	82.5	82.0/53.2

**Table 5:** Best and worst classified classes in the most accurate map by each classifier (MT, multi-temporal).

Classifier	Hraniční Meadow Peat Bog		Úpské Peat Bog (MT)	
	Class	F1-score	Class	F1-score
All classifiers	Vaccinium myrtillus	0.0	Vaccinium myrtillus	0.0
RF	Picea abies	96.6	Pinus mugo	97.8
	Pinus mugo	96.1	Eriophorum angustifolium	95.5
	Trichophorum cespitosum	91.1	Trichophorum cespitosum	92.4
	Sphagnum spp.	41.9	Vaccinium uliginosum	79.3
	Unidentified moss	26.7	Molinia caerulea	74.8
	Carex limosa	13.4	Sphagnum spp.	19.3
GBC	Water and mud	99.8	Juncus spp.	98.2
	Pinus mugo	99.0	Pinus mugo	98.0
	Picea abies	96.8	Trichophorum cespitosum	93.0
	Unidentified moss	35.8	Eriophorum angustifolium	74.1
	Carex limosa	23.7	Molinia caerulea	73.2
	Empetrum spp.	17.4	Sphagnum spp.	31.8
XGB	Pinus mugo	99.0	Pinus mugo	98.5
	Picea abies	96.8	Eriophorum angustifolium	94.1
	Trichophorum cespitosum	92.7	Trichophorum cespitosum	94.1
	Vaccinium uliginosum	37.7	Picea abies	81.2
	Dry vegetation	29.5	Molinia caerulea	76.1
	Carex limosa	26.4	Sphagnum spp.	38.6
MLP	Pinus mugo	93.4	Pinus mugo	98.7
	Picea abies	91.3	Vaccinium uliginosum	91.1
	Molinia caerulea	86.0	Calluna vulgaris	90.6
	Unidentified moss	18.8	Eriophorum angustifolium	74.0
	Sphagnum spp.	12.8	Juncus spp.	43.4
	Carex limosa	6.1	Sphagnum spp.	19.0
MLP ens.	Picea abies	93.3	Pinus mugo	99.2
	Pinus mugo	91.6	Vaccinium uliginosum	93.4
	Trichophorum cespitosum	88.5	Calluna vulgaris	92.8
	Sphagnum spp.	23.6	Nardus stricta	81.3
	Unidentified moss	15.3	Juncus spp.	72.1
	Carex limosa	12.9	Sphagnum spp.	21.1

**Table 6:** Mean contribution (%) of each feature category to global SHAP and LIME importance scores at both study sites.

Feature category	Úpské Peat Bog SHAP	Úpské Peat Bog LIME	Hraniční Meadow Peat Bog SHAP	Hraniční Meadow Peat Bog LIME
Spectral and CHM	47.3	38.7	34.5	21.5
GLCM	32.0	36.7	27.8	26.2
GLSZM	20.7	24.6	17.0	21.3
GLDM	–	–	20.8	31.0

**Table 7:** Results of ensembling (OA, weighted F1-score) and overlay analysis.

Dataset	OA (%)	F1-score (weighted/macro, %)	Agreement for overlay analysis (%)		
			At least 2 classifiers	At least 3 classifiers	4 classifiers
Úpské Peat Bog (MT)	94.2	94.0/79.2	99.4	86.6	68.7
Hraniční Meadow Peat Bog	89.8	88.7/55.6	95.0	78.9	–

For Úpské Peat Bog the ensemble consisted of RF, GBC, XGBoost and an ensemble of 10 MLPs, and for the Hraniční Meadow Peat Bog the ensemble only contained RF, GBC and XGBoost.

Of the two orthomosaics acquired at the Úpské Peat Bog, the imagery from 10 July yielded consistently higher classification accuracy (by several percentage points) compared to the 7 August dataset. Consequently, results from single-temporal classification are reported for the 10 July orthomosaic only. The texture testing results for Úpské Peat Bog are also reported for the single-temporal data from 10 July.

### 3.1 Texture testing results

The cross-validation average results for the Úpské Peat Bog and the Hraniční Meadow Peat Bog are summarized in **Tables 8**, **9**, respectively. At both sites, the inclusion of texture features improved classifier performance across all tested algorithms.

**Table 8:** Results of texture testing on the Úpské Peat Bog (cross-validation average F1-scores).

Dataset	RF	GBC	MLP	XGB
Original MS image	81.93	81.68	82.77	82.29
MS+GLCM	82.95	**83.75**	83.83	84.78
MS+GLDM	82.47	83.39	83.82	84.52
MS+GLSZM	80.39	81.83	79.76	82.84
MS+GLCM+GLDM	82.75	**83.78**	**84.26**	84.77
MS+GLCM+GLSZM	**83.21**	**83.75**	**84.24**	**85.06**
MS+GLDM+GLSZM	82.73	83.00	83.28	84.78
MS+all textures	83.05	83.23	83.19	85.02

The bold values are the best results (or very close to the best in case of more bold values in a column).

**Table 9:** Results of texture testing on the Hraniční Meadow Peat Bog (cross-validation average F1-scores).

Dataset	RF	GBC	MLP	XGB
Original MS image	82.82	82.10	72.15	83.93
MS+GLCM	84.61	85.37	77.22	86.05
MS+GLDM	84.36	84.60	72.74	86.00
MS+GLSZM	81.00	82.82	76.51	84.97
MS+GLCM+GLDM	83.27	85.14	79.92	86.26
MS+GLCM+GLSZM	**85.56**	85.73	78.45	86.50
MS+GLDM+GLSZM	84.57	85.31	**81.11**	86.97
MS+all textures	84.25	**86.01**	78.46	**87.10**

The bold values are the best results (or very close to the best in case of more bold values in a column).

For the Úpské Peat Bog, the highest or near-highest overall accuracies were achieved using a combination of GLCM and GLSZM texture features. At the Hraniční Meadow Peat Bog, however, optimal feature combinations varied by classifier. As a result, for this site, the best-performing texture feature set identified during testing was subsequently used for each classifier during the fine-tuning and final classification stages.

### 3.2 Fine tuning and classification results

The best-performing classifier at both study sites was XGBoost. At the Hraniční Meadow Peat Bog, it achieved weighted and macro F1-scores of 89.3% and 61.3% respectively (**Figure 3a**; **Supplementary Appendix D**) using all GLCM, GLDM and GLSZM texture features. At the Úpské Peat Bog, the highest weighted and macro F1-scores of 93.9% and 78.6% respectively (**Figure 3b**; **Supplementary Appendix D**) were recorded using the multi-temporal dataset (10 July and 7 August) and GLCM and GLSZM texture features. The single MLP reached the lowest accuracy at both study sites. However, on the Úpské Peat Bog, the MLP ensemble was the second most accurate, with weighted and macro F1-scores of 93.6% and 76.5% respectively. Random Forest and Gradient Boosting Classifier provided similar results, with weighted F1-scores of approximately 92% on the Úpské Peat Bog and 87% on the Hraniční Meadow Peat Bog. The corresponding optimal hyperparameters are provided in **Table 10**. On the Úpské Peat Bog, similar hyperparameters achieved best results on the multi-temporal and single-temporal datasets, while on the Hraniční Meadow Peat Bog the grid-search results differed. These hyperparameters improved accuracy metrics by a few percentage points compared to the default settings and were used in the final evaluation. Overall accuracies and F1-scores for the best models are summarized in **Table 4**.

**Table 10:** Best hyperparameters used in final evaluation.

Classifier	Hyperparameter	Úpské Peat Bog (MS)	Úpské Peat Bog (MT)	Hraniční Meadow Peat Bog
RF	n-tree	200	200	500
	max. depth	30	20	30
	min. samples to split	2	2	20
	min. samples in leaf	1	2	4
	m-try	9	9	9
GBC	n-tree	500	500	100
	learning rate	0.01	0.01	0.1
	max. depth	7	7	7
	min. samples to split	2	2	10
	min. samples in leaf	1	1	2
XGB	n-tree	500	500	100
	learning rate	0.2	0.2	0.2
	max. depth	3	3	3
	subsample	0.5	0.5	0.7
	lambda	0.5	0.5	1
MLP	hidden layer sizes	1 layer of 50 n.	1 layer of 50 n.	2 layers of 50 n.
	max. iterations	200	200	200
	alpha	0.00001	0.00001	0.0001

Across both sites, several classes were classified with F1-scores above 90% by most classifiers. The highest and lowest class-level F1-scores are presented in **Table 5**, with full results provided in **Supplementary Appendix B**. Common species, such as *Pinus mugo* and *Trichophorum cespitosum*, were generally classified most accurately, whereas classes with limited training samples, e.g., *Carex limosa* on the Hraniční Meadow Peat Bog or *Vaccinium myrtillus* on both sites, exhibited lower performance. There were some cases of well-classified classes with a small number of data samples, most notably *Eriophorum angustifolium* and *Juncus* spp. on the Úpské Peat Bog. In most cases, classifiers agreed on the best- and worst-performing classes; however, exceptions occurred, such as *Eriophorum angustifolium*, which was poorly classified by GBC but among the best classified by XGB. There were significantly more low-performing classes on the Hraniční Meadow Peat Bog, which is consistent with the lower weighted F1-score. On Úpské Peat Bog, the vegetation maps, generated by the three tree-based classifiers, look visually similar, but the map generated by the MLP ensemble differs more. On Hraniční Meadow Peat Bog, the GBC map looks more similar to the map generated by the MLP ensemble than the ones produced by the other tree-based classifiers (Appendix D, figures D.3, D.4).

### 3.3 Feature importance and interpretability

On both sites, SHAP and LIME approaches agreed on the importance of feature categories. Spectral and CHM features contributed 47.3% (SHAP) and 38.7% (LIME) of total importance at Úpské Peat Bog, while GLCM and GLDM together accounted for 48.6% (SHAP) and 57.2% (LIME) at Hraniční Meadow Peat Bog (**Table 6**). Both methods ranked the CHM feature first at each site; SHAP and LIME means were 1.20 and 0.071 at Úpské Peat Bog and 1.33 and 0.086 at Hraniční Meadow Peat Bog, respectively. The next four global features at Úpské Peat Bog were the spectral features, followed by three GLCM mean features from NIR, RE and Green bands. At Hraniční Meadow Peat Bog, ranks 2–8 were a mix of spectral bands, GLCM mean features and GLDM homogeneity features. At the class level, *Molinia caerulea* showed the highest single-class value at Úpské Peat Bog (CHM, SHAP = 4.17), while the CHM SHAP values for *Picea abies* (3.65) and *Trichophorum cespitosum* (3.44) at Hraniční Meadow Peat Bog ranked second and third. Detailed class-level SHAP and LIME rankings for all vegetation classes at both study sites are provided in **Supplementary Appendix F** (Tables F.1 and F.2).

### 3.4 Ensembling results

**Figure 4:**
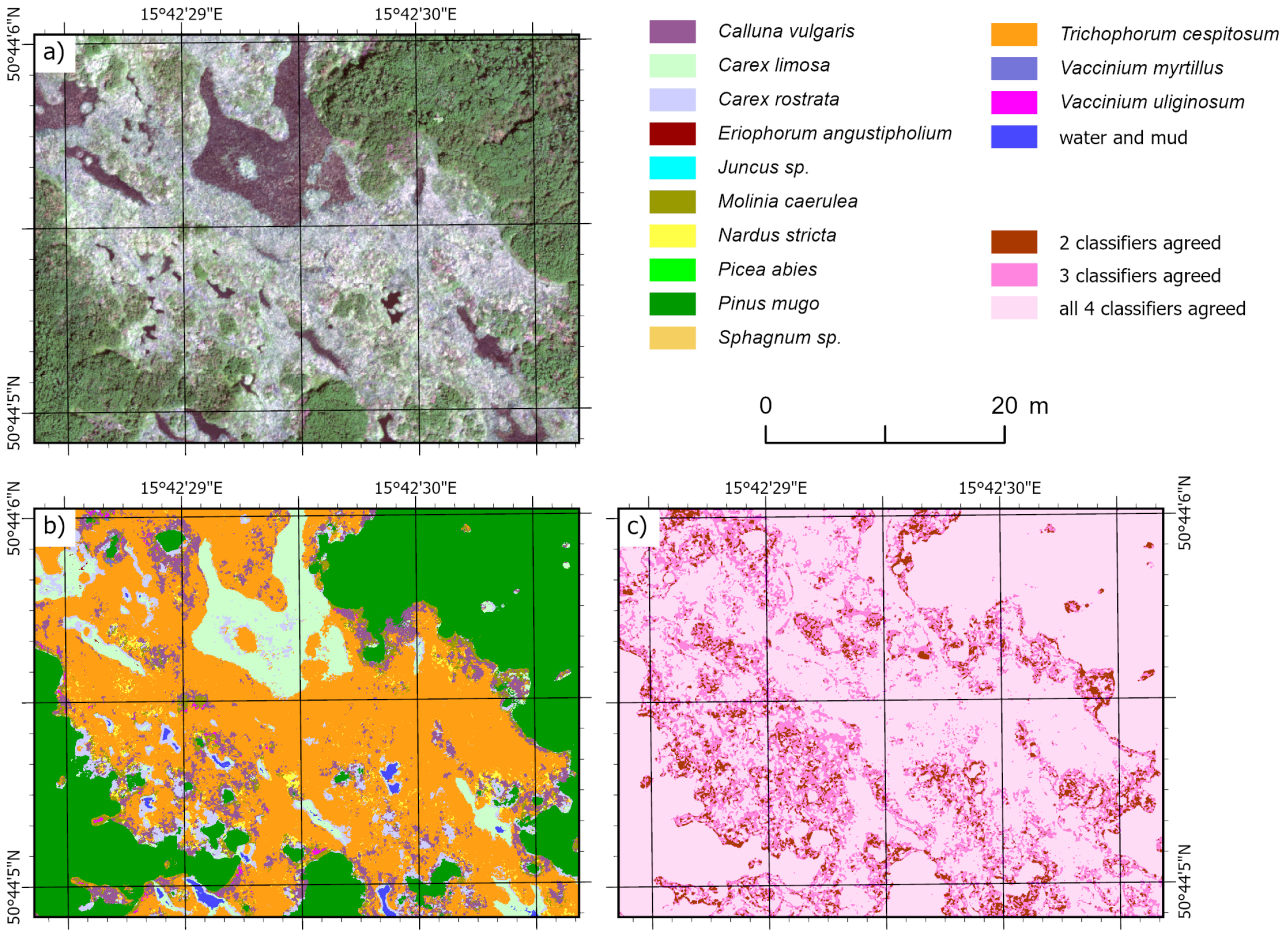
Comparison of the original UAV orthomosaic **(a)**, the ensemble classification **(b)** and the classification agreement **(c)** for the Úpské Peat Bog.

At the Úpské Peat Bog, the ensemble model slightly outperformed the best individual model, achieving weighted and macro F1-scores of 94.0% and 79.2% respectively and an overall accuracy of 94.2% on the multi-temporal dataset (**Figure 3b**; **Supplementary Appendix D**). The accompanying consistency map revealed high inter-model agreement inside large areas of homogeneous vegetation and lower inter-model agreement typically observed at the edges of the vegetation patches (**Figure 4**). All four classifiers agreed on 68.7% of the map; at least three classifiers agreed on 86.6% (**Table 7**).

At the Hraniční Meadow Peat Bog, none of the individual models reached the target accuracy threshold of 90%. However, for comparative analysis, an ensemble of the three best-performing models (RF, GBC, XGBoost) was constructed. While its accuracy was lower than that of the best single model (F1-score 88.7%), the resulting agreement map exhibited similar spatial patterns to those observed at the Úpské Peat Bog, with reduced consistency along patch boundaries. All three classifiers agreed on 78.9% of the map (**Table 7**).

## 4 Discussion

The classification results show that the proposed method enables accurate and spatially detailed mapping of species composition in alpine peat bog vegetation. This level of detail allows vegetation patterns and their changes to be interpreted as indicators of underlying hydrological and ecological conditions, confirming the suitability of UAV data for long-term monitoring. To our knowledge, this study represents one of the first systematic multi-classifier evaluations combined with a comprehensive assessment of three texture feature matrix types in an alpine peat bog context, evaluated under identical conditions across two ecologically distinct sites. On the Úpské Peat Bog, a weighted F1-score of 94.0% was achieved, which is remarkable given the site’s complexity and extent, while slightly lower accuracy was obtained for the Hraniční Meadow Peat Bog (weighted F1-score 89.3%). These results are comparable to or exceed most wetland vegetation mapping studies (Cao et al., 2018; Gonzalez-Perez et al., 2022; Pande-Chhetri et al., 2017) and alpine vegetation mapping in the Krkonoše Mountains (Marcinkowska-Ochtyra et al., 2018; Zagajewski et al., 2021). Although Kupková et al. (2023) reported higher accuracy (OA 95.9%) for grass species classification in the Krkonoše Mountains, their study considered only seven species in a less complex tundra grassland. In contrast, our approach integrates CHM and texture features to map 14 peat bog classes in a more heterogeneous environment, improving Úpské Peat Bog results by 7.5% compared to Kupková et al. (2020).

XGBoost was the best-performing classifier at both sites, consistent with previous studies (Zhang et al., 2019; Yoon and Jung, 2024; Zhen et al., 2024). A 10-MLP ensemble achieved comparable results at the Úpské Peat Bog but performed substantially worse at the Hraniční Meadow Peat Bog (weighted F1-score 82.0%), indicating limited robustness. A single MLP also exceeded a weighted F1-score of 90% at Úpské Peat Bog but was the weakest model at Hraniční Meadow Peat Bog (weighted F1-score 81.5%). RF and GBC showed similar performance, both slightly below XGBoost, although GBC required substantially longer tuning time, which limits its operational applicability. Additional approaches were tested but not retained. Convolutional Neural Networks (Sothe et al., 2020; Yang et al., 2020) failed to learn rare classes, likely due to limited data, while Support Vector Machines required computationally demanding fine tuning. Both were therefore excluded. Object-based image analysis was also evaluated as an alternative classification strategy. However, it achieved lower accuracy than the pixel-based approach, likely because the substantially smaller number of image objects, compared with individual pixels, reduced the effective number of samples available for model training and testing in the highly heterogeneous peatland environment. In addition, the pixel-based workflow was more straightforward to implement and less dependent on segmentation settings and specialized OBIA software.

While weighted F1-score and overall accuracy provide useful overall metrics, they are influenced by dominant classes. A more comprehensive evaluation requires class-level and macro-averaged F1-scores. Most classes were mapped with high accuracy at the Úpské Peat Bog (best macro F1-score 78.6%), enabling reliable representation of both dominant and rare species, whereas performance at Hraniční Meadow Peat Bog was lower (best macro F1-score 61.3%). Key peat bog species such as *Carex limosa*, *Trichophorum cespitosum*, and *Calluna vulgaris* achieved best F1-scores above 90%. At Úpské Peat Bog, even rare classes such as *Eriophorum angustifolium* (95.5%) and *Juncus* spp. (98.9%) were mapped accurately. In contrast, six classes at Hraniční Meadow Peat Bog had F1-scores below 60%, compared to only two at Úpské Peat Bog, largely reflecting limited training data (<10 polygons, <1000 samples). *Carex limosa* showed strong site-dependent performance (>80% at Úpské Peat Bog vs. 26% at Hraniční Meadow Peat Bog), and overall variability across classifiers was higher at Hraniční Meadow Peat Bog.

*Vaccinium myrtillus* remained undetected at both sites (F1-score 0.0), primarily due to critically limited training data and partly due to low spectral separability from *Vaccinium uliginosum*, confirmed by the lowest Jeffries-Matusita values in both datasets (1.48 at Úpské, 1.53 at Hraniční). In the confusion matrices, this class was most frequently misclassified as *Pinus mugo*, which likely reflects class imbalance rather than genuine spectral similarity. If sufficient additional sampling cannot be obtained given its sparse field occurrence, merging the two Vaccinium species into a single class could represent the most practical remedy. However, class merging does not always improve overall performance; in this case it was tested and resulted in a decrease in overall accuracy and F1-scores for several individual classes, which is why the original class structure was retained. *Sphagnum* spp. also showed consistently low F1-scores despite comparatively adequate training data, indicating a fundamentally spectral rather than data quantity problem. Merging this class with any other class would be ecologically unjustifiable given the distinct hydrological indicator roles, and improved discrimination would likely require additional features.

Substantial inter-model variability was observed for several classes. Differences were most pronounced between tree-based models (RF, GBC, XGB) and neural networks (MLPs). For example, tree-based models consistently classified *Juncus* spp. at Úpské Peat Bog with high accuracy (>92%), whereas MLPs performed worse (best F1-score 82.6%). Conversely, MLPs outperformed tree-based models for *Vaccinium uliginosum* at both sites (93.4% *vs*. 86.1% and 88.5% *vs*. 61.5%). These results highlight the importance of comparing multiple robust classifiers, as individual models may underperform for specific classes. For instance, in the multi-temporal Úpské Peat Bog dataset, RF and GBC achieved similar overall performance (92.5% and 92.3%) but differed substantially at class level: RF performed better for *Eriophorum angustifolium* (95.5% *vs*. 74.1%) and *Picea abies* (86.3% *vs*. 78.9%), whereas GBC was superior for *Juncus* spp. (98.2% *vs*. 84.6%). **Table 11** compares the best F1-scores obtained in this study with previous studies.

**Table 11:** Comparison of F1-scores for different species with previous studies.

Species	Location	Best F1-score (%)	Study	Best F1-score (%)
*Trichophorum cespitosum*	Úpské p.b.	94.1	Kupková et al., 2017	77.0
*Nardus stricta*	Úpské p.b.	85.9	Kupková et al., 2023	94.7
*Calluna vulgaris*	Úpské p.b.	92.8	Simpson et al., 2024	84.8
*Picea abies*	Hraniční m.	96.8	Zagajewski et al., 2021	91.7
*Molinia caerulea*	Úpské p.b.	83.2	Kupková et al., 2023	99.2
*Pinus mugo*	Úpské p.b.	99.2	Kupková et al., 2017	99.7
*Pinus mugo*	Hraniční m.	99.6		

Lower accuracy in some classes is primarily explained by limited data availability and variable data quality. The Hraniční Meadow Peat Bog dataset contained fewer field observations and only single-temporal imagery, reducing the available information. Ground-truth quality may also vary among classes, and data acquisition is prone to measurement and preprocessing errors (Morrison et al., 2020). Not all classes with limited samples performed poorly, but all classes with sufficient training data were classified well. Common species such as *Trichophorum cespitosum*, *Pinus mugo*, *Nardus stricta*, and *Calluna vulgaris* were well represented and consistently mapped with high accuracy. For rare species, high accuracy was achieved only when distinctive spectral characteristics were present. For example, *Juncus* spp. reached an F1-score of 94.4% despite limited training data, likely due to its characteristic orange coloration during the vegetation season. A similar pattern was observed for *Eriophorum angustifolium* (F1-score 97.0%), which turns red in summer. In contrast, species lacking a distinct spectral signature, such as *Vaccinium* or *Sphagnum* spp., were poorly classified even with comparable sample sizes.

An unexpected result was the relatively low accuracy (<90%) of the “Water and mud” class at the Úpské Peat Bog. Previous studies (Wen et al., 2025; La Salandra et al., 2023) indicate that water is typically classified with high accuracy, but this becomes more challenging in wetlands, where shallow water is often mixed with vegetation such as *Carex* and *Eriophorum*. This increases spectral ambiguity and highlights the importance of precise and consistent ground-truth data. If training samples include sparse aquatic vegetation, some pixels may represent only shallow water, leading to confusion in feature space. Initially, water and mud were treated as separate classes, but due to poor separability they were merged. This likely reflects the similar spectral response of wet mud and shallow, sediment-rich water.

Field collection of GNSS data remains a major limitation. In addition to difficult accessibility and logistical constraints, identifying sufficiently large homogeneous patches for sampling is challenging in heterogeneous peat bogs. Improving ground-truth quality, particularly for rare classes, would likely enhance classification performance and support wetland management. Dataset expansion using semi-supervised approaches (Chen et al., 2023; Wang et al., 2023; Farsad Layegh et al., 2022) may offer potential but still depends on the availability of high-quality field data. Class imbalance also remains an issue. Although stratified sampling reduces discrepancies between training and overall class distributions, final class proportions still differed from initial estimates (**Supplementary Appendix C**).

Texture features improved classification accuracy in both datasets, in agreement with previous studies (Michez et al., 2016; Mohammadpour et al., 2022; Erdem and Bayrak, 2023). The combination of texture features proved more important than individual feature sets. At the Úpské Peat Bog, GLCM provided the best single-feature improvement (1–2%), while combining GLCM with GLSZM or GLDM further increased accuracy (by 0–0.5%). However, combining all three reduced performance, and the final model therefore used GLCM and GLSZM only. At the Hraniční Meadow Peat Bog, improvements were less consistent, ranging from 3–9%, with optimal feature combinations varying by classifier. This indicates that although texture features enhance classification, no universally optimal configuration exists, and dataset-specific testing is required. These results highlight the importance of spatial context for capturing fine-scale vegetation patterns in heterogeneous peatland environments.

The feature importance analysis reveals that canopy height, captured by the CHM band, was the single strongest discriminator at both sites (global mean SHAP = 1.20 and 1.33), underlining that structural differentiation, rather than spectral contrast alone, drives classification performance in these spectrally similar peatland communities. This finding is consistent with Hamedianfar et al. (2025), who similarly identified DSM as the dominant SHAP feature across all ensemble classifiers in UAS-based peatland vegetation mapping, confirming the central role of canopy structure in discriminating spectrally similar peatland classes. The effect is particularly pronounced for *Picea abies* (3.65), *Trichophorum cespitosum* (3.44), and *Molinia caerulea* (4.17), whose vertical stature sets them apart from the low, mat-forming communities with which they co-occur. The dominance of texture features at Hraniční Meadow Peat Bog (GLCM + GLDM: 48.6% SHAP combined) compared to Úpské Peat Bog (32.0% GLCM SHAP), where multi-temporal spectral bands contributed more, is consistent with Hraniční Meadow Peat Bog being captured in a single flight: without phenological contrast between dates, the classifier relies instead on fine-scale canopy texture to separate structurally similar low-growing communities. At the class level at Úpské Peat Bog, *Nardus stricta* was classified primarily via GLCM NIR mean (|SHAP| = 2.37), suggesting its classification depends on the textural regularity of its dense, fine-leaved mat rather than on unique spectral reflectance, while *Carex rostrata* on the Úpské Peat Bog was also distinguished by GLCM and GLSZM texture features ahead of spectral ones, consistent with its coarser and more spatially variable canopy structure. However, with *Carex rostrata* the same effect cannot be seen at Hraniční Meadow Peat Bog, where the CHM was the most important feature. Because this effect does not play out in other species, this distinction suggests important differences in vegetation structure between the two study sites. The agreement between SHAP and LIME rankings across both sites confirms that these feature hierarchies are stable and not an artefact of any single explanation method.

Multi-temporal UAV data improved classification accuracy, consistent with previous studies (Kupková et al., 2023). However, unlike texture features, multi-date acquisition is more time-consuming and logistically demanding. Its applicability in future KRNAP projects and similar environments therefore remains under consideration. From a classification perspective, more frequent acquisitions across the vegetation season are desirable, as they capture species-specific phenological and spectral variability. At the same time, this approach introduces limitations. In our study, the accuracy of the “Water and mud” class slightly decreased in the multi-temporal dataset, likely due to changes in water levels between acquisition dates. Differences in illumination conditions, vegetation stage, and sensor setup among orthomosaics may further increase variability in model performance. Notably, the orthomosaic from 7 August did not achieve a weighted F1-score above 90% and a macro F1-score above 70%, which may reflect suboptimal phenological timing, atmospheric conditions, or sensor differences. It should also be noted that the two Úpské acquisitions involved different sensors; residual inter-sensor biases in spectral response may have contributed to the lower August accuracy alongside phenological and atmospheric factors, and their relative contributions cannot be disentangled. The multi-temporal classification results should therefore be interpreted with this limitation in mind. Nevertheless, combining multiple dates enriched the feature space and helped identify the most suitable acquisition timing for single-date classification, depending on species development and seasonal color variation. More broadly, the classifiers developed in this study were trained exclusively on summer imagery, when many peat bog species exhibit their most distinctive spectral characteristics, a factor that likely contributed to the high accuracy achieved for spectrally distinctive species such as *Juncus* spp. and *Eriophorum angustifolium*. Outside the summer period, canopy structure and spectral signatures undergo substantial changes, and assessing model robustness across seasons and identifying the optimal acquisition window for individual species could represent an important direction for future work. It should also be noted that in alpine mountain environments such as the Krkonoše Mountains, the practical monitoring window is substantially constrained, because snow cover typically persists from October to June, and early season acquisitions are further limited by underdeveloped vegetation and unfavorable weather conditions in spring. This effectively restricts reliable UAV-based vegetation monitoring to the core summer months.

Recent studies have demonstrated that combining multispectral imagery with hyperspectral data, LiDAR point clouds, or polarimetric SAR can substantially improve species-level classification accuracy in complex wetland environments such as mangroves and karst wetlands (Yao et al., 2025; Guo et al., 2023; Fu et al., 2023). Multi-temporal satellite time series have similarly shown promise for aquatic vegetation classification in temperate wetlands (Piaser and Villa, 2023). While such data fusion approaches represent a clear avenue for further accuracy improvements, they were beyond the scope of the present study, which deliberately focused on multispectral UAV imagery as a widely accessible and cost-effective platform for long-term alpine peatland monitoring. Incorporating additional data sources such as hyperspectral or LiDAR data in future work could particularly benefit the classification of spectrally similar low-growing species such as *Sphagnum* spp. and *Vaccinium myrtillus*, which proved most challenging under the current data configuration.

An important contribution of this study is the use of an ensemble-based approach to map spatial classification agreement across vegetation patterns. While classification metrics indicate excellent overall performance, spatial variability in classification agreement provides additional insight that cannot be captured by global accuracy metrics alone. Despite its importance, such agreement mapping is still rarely implemented in remote sensing studies, making this approach notably innovative. The resulting maps can support targeted field validation by identifying areas of high and low inter-model agreement and serve as a complementary spatial layer for assessing the consistency of predicted vegetation classes, thereby improving interpretation of species distribution and vegetation structure. This makes the ensemble-derived agreement maps a practical and robust tool for interpreting and applying vegetation mapping and classification results in an ecological context.

One of the aims of our study was to develop a methodological framework supporting long-term, reliable monitoring of vegetation dynamics in alpine peat bog ecosystems. Based on the results of this study, it is evident that peat bogs can be mapped with high spatial detail to support such monitoring following these steps:

1. Data acquisition phase:
  - The UAV image data should be captured under homogeneous lighting conditions (Dandois et al., 2015) with a spatial resolution of 5 cm or better (Simpson et al., 2024) and minimum front and side overlaps of 70–85% (Dandois et al., 2015) to ensure data robustness and usefulness. The UAV should be equipped with an RTK (Real-Time Kinematic) system for real-time positioning accuracy or a PPK (Post-Processed Kinematic) system for post-processing-based accuracy enhancement.
  - The ground-truth botanical data coordinates should be collected using precise GNSS equipment with centimeter-level accuracy, which is critical for the heterogeneous and complex peat bog environment.
  - It is crucial not to underestimate the amount of ground-truth data collected, especially for rare classes. A minimum of 6–10 polygons and 1000 pixels per species are recommended, depending on class abundance and spectral distinctiveness. For common classes, a much higher number (dozens to hundreds of polygons, depending on the size of the area of interest) is advisable.
2. Data preprocessing phase
  - Imagery should be processed using photogrammetric software such as Agisoft Metashape or Pix4D.
  - Before classification, additional features should be incorporated, such as canopy height models and texture features derived from GLCM, GLDM, or GLSZM matrices. These features can substantially (by 5–10%) improve classification accuracy and subsequent vegetation mapping quality.
  - Individual polygons should not be split arbitrarily between training and validation/test sets during preprocessing. Instead, a stratified sampling approach should be used to ensure proper class representation in the dataset, based on observed proportions in the field (as indicated by an initial classification).
3. Data analysis phase
  - Cross-validation alongside an independent test dataset should be used to avoid overfitting and to ensure robust accuracy estimates.
  - The F1-score weighted by class abundance can be used as the primary metric of classification accuracy, but also the unweighted (macro-averaged) F1-score and F1-scores for individual classes should be thoroughly examined.
  - The XGBoost classifier is recommended for its reliability, performance, and flexibility; however, exploring other classifiers and combining them in an ensemble may further improve results and enhance vegetation mapping robustness.
  - The spatial agreement between multiple strong classifiers can be used as an indicator of consistency in the resulting vegetation maps, supporting their interpretation for monitoring purposes.

This approach is based on the findings of our study as well as established recommendations in the scientific literature, and it ensures high accuracy for future vegetation monitoring. In the field, however, it may be difficult to consistently follow all recommended steps, especially during the data collection phase. In mountain environments, optimal lighting conditions for UAV imaging are rare, and homogeneous illumination cannot be guaranteed. Limitations may also affect the collection of reference data, for example, when certain classes are too rare to allow for the collection of homogeneous samples. Nevertheless, for reliable vegetation mapping supporting ecological monitoring, the recommended steps should be followed to the greatest extent possible.

## 5 Conclusion

This study demonstrates that species-level vegetation patterns in alpine peat bogs can be reliably captured and interpreted using UAV data, machine learning, texture-based features and ground-truth botanical data. Focusing on two ecologically distinct sites in the Krkonoše Mountains, the Úpské and Hraniční Meadow Peat Bogs, we systematically compared four classifiers and evaluated texture features derived from GLCM, GLDM, and GLSZM matrices. The proposed workflow achieved very high accuracy (weighted and macro F1-scores up to 94% and 79% respectively) while enabling detailed mapping of species composition in heterogeneous and complex environments. XGBoost consistently provided the best performance, confirming its robustness for species-level mapping. Ensemble approaches, including MLP ensembles, in some cases performed comparably, indicating that per-pixel neural networks may represent a viable alternative under specific conditions.

A key contribution of this study is the systematic integration of texture features, which improved classification accuracy and enhanced the representation of fine-scale spatial variability in vegetation structure. While GLCM performed best as a single feature set, combining it with GLSZM further improved results, with optimal configurations varying by classifier and dataset. The study also introduces an ensemble-based agreement mapping approach, providing spatially explicit information on classification consistency. This method supports targeted field validation and improves ecological interpretation of vegetation patterns. In addition, multi-temporal UAV data proved beneficial for capturing species-specific phenological variability, although practical limitations related to acquisition effort and data consistency highlight the need for careful survey design.

We propose a transferable, high-performing and field-validated methodological framework integrating UAV multispectral data, texture features, canopy height models, ground-truth botanical data, and robust model validation. This study advances UAV-based species-level vegetation mapping and enables high-resolution monitoring of peatland vegetation dynamics, where species composition and its changes serve as important indicators of ecosystem state, improving the detection and interpretation of ecosystem dynamics. It therefore represents a practical tool for conservation and adaptive management of these vulnerable alpine peatland ecosystems.

## Data Availability

The raw data supporting the conclusions of this article will be made available by the authors, without undue reservation.
