# Supplementary material for: Advancing species-level vegetation mapping of alpine peat bogs using UAV imagery, machine learning and texture features
Source: Front Plant Sci. 2026 Jun 29;17:1841696. doi: 10.3389/fpls.2026.1841696 (PMC13357729; doi:10.3389/fpls.2026.1841696)
Supplement: Supplementary file 1 [file Supplementaryfile1.docx]

# Appendices

**Appendix A. Grid values used in hyperparameter tuning of each model.**

Table A.1. Grid values used in hyperparameter tuning of each model.

| **Classifier** | **Hyperparameter** | **Grid values** |
| --- | --- | --- |
| **RF** | n-tree | 50, 100, 150, 200, 300, 400, 500, 750 |
|  | max. depth | 2, 3, 5, 7, 10, 15, 20, 25, 30, 40, None |
|  | min. samples to split | 1, 2, 3, 5, 7, 10, 12, 15, 20, 30 |
|  | min. samples in leaf | 1, 2, 3, 4, 5, 7, 10 |
|  | m-try | 6, 7, 8, 9, 10, 11, 12 |
| **GBC** | n-tree | 50, 100, 150, 200, 300, 400, 500, 750 |
|  | learning rate | 0.0001, 0.001, 0.01, 0.1, 0.2, 0.5, 1 |
|  | max. depth | 2, 3, 5, 7, 10, 15, 20, 25, 30, 40, None |
|  | min. samples to split | 1, 2, 3, 5, 7, 10, 12, 15, 20, 30 |
|  | min. samples in leaf | 1, 2, 3, 4, 5, 7, 10 |
| **XGB** | n-tree | 50, 100, 150, 200, 300, 400, 500, 750 |
|  | learning rate | 0.0001, 0.001, 0.01, 0.1, 0.2, 0.5, 1 |
|  | max. depth | 2, 3, 5, 7, 10, 15, 20, 25, 30, 40, None |
|  | subsample | 0.3, 0.5, 0.7, 1 |
|  | lambda | 0.1, 0.2, 0.5, 0.7, 1, 2, 5 |
| **MLP** | hidden layer sizes | (25), (25,25), (50), (50,50), (100), (100,100), (200), (200,200), (500), (500,500) |
|  | max. iterations | 100, 200, 500 |
|  | alpha | 0.000001, 0.00001, 0.0001, 0.001, 0.01, 0.1, 1 |

**Appendix B. Complete testing results including individual classes.**

Table B.1. Individual classes F1-score for the Úpské Peat Bog

|  | **Single-temporal** | | | | | **Multi-temporal** | | | | | |
| --- | --- | --- | --- | --- | --- | --- | --- | --- | --- | --- | --- |
|  | RF | GBC | XGB | MLP | MLP ens. | RF | GBC | XGB | MLP | MLP ens. | Big ens. |
| **Overall accuracy** | 88.5 | 88.5 | 91.0 | 87.2 | 89.1 | 92.5 | 92.3 | 94.2 | 91.1 | 93.7 | 94.2 |
| **F1 weighted** | 88.5 | 88.4 | 90.9 | 86.9 | 88.9 | 92.2 | 92.1 | 93.9 | 91.1 | 93.6 | 94.0 |
| **macro F1** | 71.0 | 71.4 | 76.7 | 68.8 | 70.8 | 74.8 | 74.5 | 78.6 | 71.2 | 76.5 | 79.3 |
| *Calluna vulgaris* | 82.7 | 88.4 | 88.0 | 66.2 | 85.5 | 83.2 | 87.2 | 91.9 | 90.6 | 92.8 | 93.6 |
| *Carex limosa* | 71.5 | 77.6 | 79.3 | 74.2 | 79.5 | 85.2 | 87.2 | 92.2 | 83.8 | 88.6 | 92.1 |
| *Carex rostrata* | 63.6 | 65.1 | 70.4 | 67.3 | 72.3 | 80.2 | 81.1 | 86.9 | 78.6 | 84.0 | 86.4 |
| *Eriophorum angustifolium* | 81.0 | 93.9 | 95.0 | 84.2 | 90.7 | 95.5 | 74.1 | 94.1 | 74.0 | 82.6 | 97.0 |
| *Juncus spp.* | 92.5 | 98.9 | 95.8 | 56.6 | 82.6 | 84.6 | 98.2 | 90.3 | 43.4 | 72.1 | 94.4 |
| *Molinia caerulea* | 72.8 | 76.2 | 82.0 | 70.9 | 78.1 | 74.8 | 73.2 | 76.1 | 78.8 | 83.2 | 76.4 |
| *Nardus stricta* | 81.1 | 80.7 | 85.1 | 78.8 | 74.3 | 85.9 | 85.5 | 84.0 | 78.5 | 81.3 | 86.3 |
| *Picea abies* | 84.3 | 80.7 | 82.7 | 84.1 | 80.3 | 86.3 | 78.9 | 81.2 | 84.8 | 92.0 | 81.9 |
| *Pinus mugo* | 98.4 | 97.5 | 98.3 | 97.9 | 98.3 | 97.8 | 98.0 | 98.5 | 98.7 | 99.2 | 98.5 |
| *Sphagnum spp.* | 34.2 | 2.6 | 33.7 | 39.0 | 1.7 | 19.3 | 31.8 | 38.6 | 19.0 | 21.1 | 37.3 |
| *Trichophorum cespitosum* | 87.5 | 85.3 | 89.9 | 81.4 | 84.5 | 92.4 | 93.0 | 94.1 | 88.1 | 91.7 | 93.8 |
| *Vaccinium myrtillus* | 0.0 | 0.0 | 0.0 | 0.0 | 0.0 | 0.0 | 0.0 | 0.0 | 0.0 | 0.0 | 0.0 |
| *Vaccinium uliginosum* | 60.3 | 73.3 | 82.9 | 75.9 | 74.5 | 79.3 | 79.4 | 86.4 | 91.1 | 93.4 | 81.6 |
| Water and mud | 84.7 | 78.7 | 90.4 | 86.2 | 88.4 | 82.2 | 75.9 | 86.0 | 87.3 | 88.6 | 86.3 |

Table B.2. Individual classes F1-score for the Hraniční Meadow Peat Bog

|  | **RF** | **GBC** | **XGB** | **MLP** | **MLP ens.** | **Big ens.** |
| --- | --- | --- | --- | --- | --- | --- |
| **Overall accuracy** | 87.7 | 87.5 | 90.2 | 83.0 | 82.5 | 89.8 |
| **F1 weighted** | 86.5 | 87.2 | 89.3 | 81.5 | 81.0 | 88.7 |
| **macro F1** | 58.5 | 55.1 | 61.3 | 55.6 | 53.2 | 55.6 |
| *Carex limosa* | 13.4 | 23.7 | 26.4 | 6.1 | 12.9 | 32.4 |
| *Carex rostrata* | 72.4 | 68.2 | 84.6 | 69.9 | 52.9 | 82.8 |
| *Empetrum spp.* | 58.3 | 17.4 | 67.6 | 62.1 | 56.1 | 30.7 |
| *Molinia caerulea* | 60.6 | 61.8 | 81.1 | 86.0 | 65.7 | 74.5 |
| *Picea abies* | 96.6 | 96.8 | 96.2 | 91.3 | 93.3 | 95.8 |
| *Pinus mugo* | 96.1 | 99.0 | 99.6 | 93.4 | 91.6 | 99.8 |
| *Sphagnum spp.* | 41.9 | 38.2 | 38.7 | 12.8 | 23.6 | 32.9 |
| *Trichophorum cespitosum* | 91.1 | 90.1 | 92.7 | 75.0 | 89.9 | 91.8 |
| *Vaccinium myrtillus* | 0.0 | 0.0 | 0.0 | 0.0 | 0.0 | 0.0 |
| *Vaccinium uliginosum* | 61.5 | 37.2 | 37.7 | 74.3 | 88.5 | 29.7 |
| Unidentified moss | 26.7 | 35.8 | 44.7 | 18.8 | 15.3 | 13.5 |
| Dry vegetation | 66.3 | 48.1 | 29.5 | 62.1 | 53.2 | 29.3 |
| Water and mud | 76.0 | 99.8 | 97.5 | 70.4 | 48.6 | 98.4 |

**Appendix C. Area proportions of individual classes according to the most accurate classification outputs**

Table C.1. Area proportions - comparison between initial classification used to calibrate stratified sampling and the final most accurate classification on Úpské Peat Bog

| **Class** | **Area proportion according to initial classification (%)** | **Area proportion according to final classification (%)** |
| --- | --- | --- |
| *Calluna vulgaris* | 5.5 | 5.4 |
| *Carex limosa* | 7.3 | 4.4 |
| *Carex rostrata* | 3.8 | 6.0 |
| *Eriophorum angustifolium* | 0.09 | 0.4 |
| *Juncus spp.* | 0.3 | 0.1 |
| *Molinia caerulea* | 3.9 | 6.0 |
| *Nardus stricta* | 22.8 | 11.0 |
| *Picea abies* | 0.2 | 0.2 |
| *Pinus mugo* | 37.6 | 42.2 |
| *Sphagnum spp.* | 0.05 | 0.09 |
| *Trichophorum cespitosum* | 15.6 | 22.4 |
| *Vaccinium myrtillus* | 0.007 | 0.1 |
| *Vaccinium uliginosum* | 1.2 | 0.3 |
| Water and mud | 1.8 | 1.3 |

Table C.2. Area proportions - comparison between initial classification used to calibrate stratified sampling and the final most accurate classification on Hraniční Meadow Peat Bog

| **Class** | **Area proportion according to initial classification (%)** | **Area proportion according to final classification (%)** |
| --- | --- | --- |
| *Carex limosa* | 5.6 | 7.4 |
| *Carex rostrata* | 6.5 | 2.3 |
| *Empetrum spp.* | 0.5 | 2.9 |
| *Molinia caerulea* | 6.0 | 1.7 |
| *Picea abies* | 12.2 | 7.1 |
| *Pinus mugo* | 50.3 | 59.1 |
| *Sphagnum spp.* | 3.6 | 1.9 |
| *Trichophorum cespitosum* | 9.9 | 14.5 |
| *Vaccinium myrtillus* | 1.5 | 0.5 |
| *Vaccinium uliginosum* | 0.6 | 0.6 |
| Unidentified moss | 1.4 | 0.4 |
| Dry vegetation | 0.3 | 0.5 |
| Water and mud | 1.6 | 1.2 |

**Appendix D. Best vegetation maps and comparison of classification outputs**

Figure D.1. Hraniční Meadow Peat Bog classified by XGBoost with weighted average F1-score of 89.3
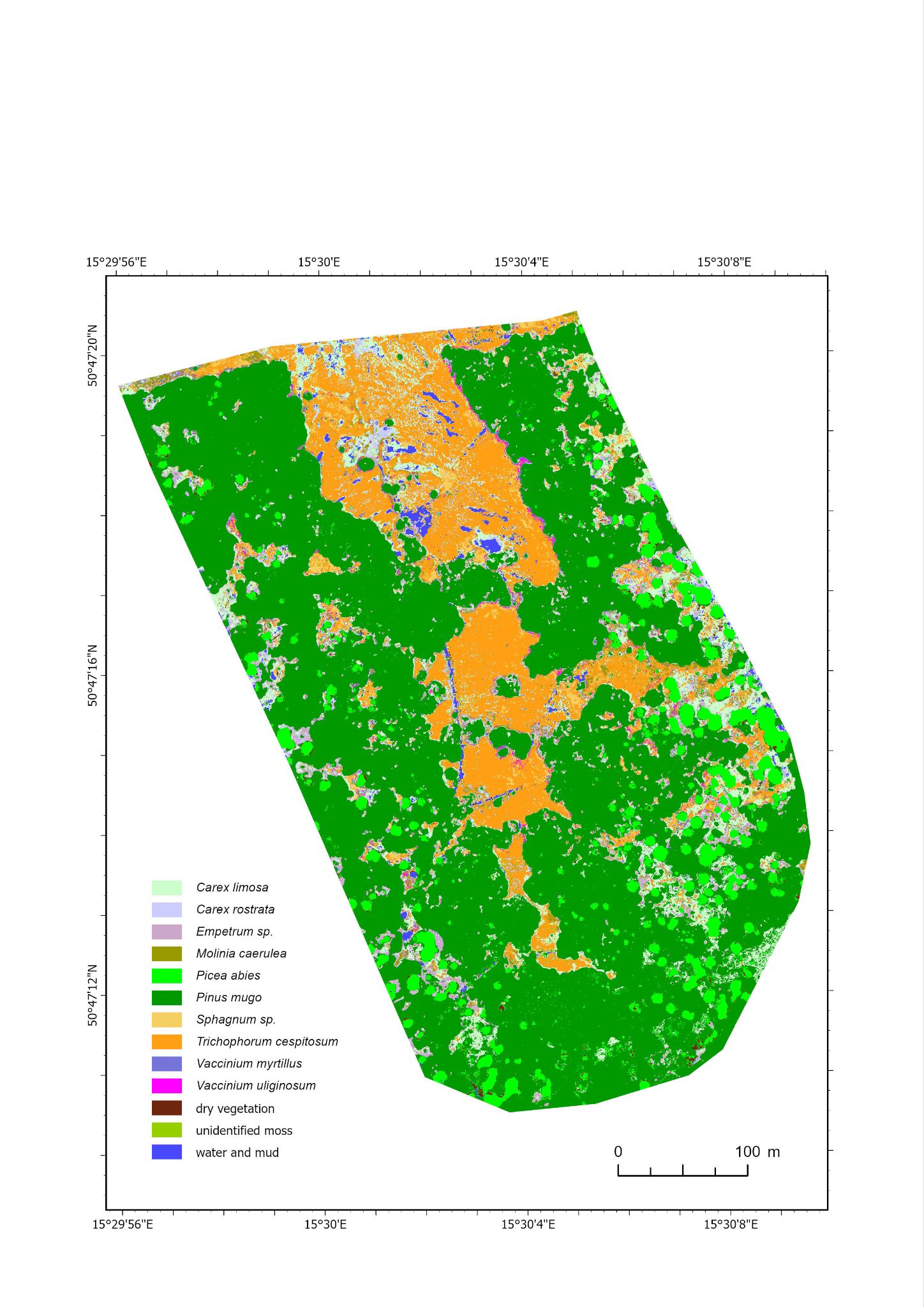


Figure D.2. Úpské Peat Bog classified by ensemble of RF, GBC, XGB and 10 MLPs with weighted average F1-score of 94.0


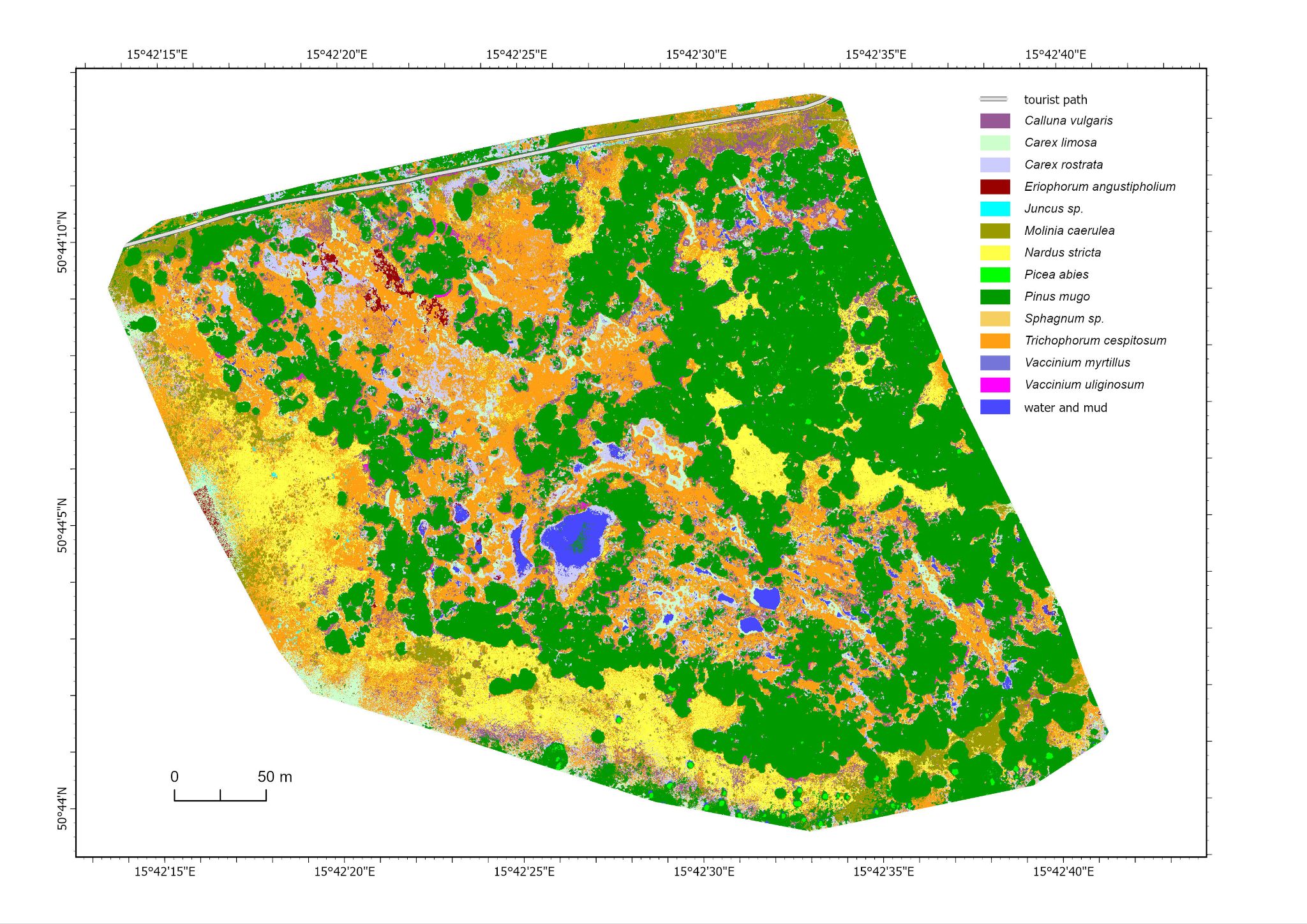


Figure D.3. Detailed comparison of classification results on Úpské Peat Bog: RGB visualisation (a), XGB (b), RF (c), GBC (d), MLP ensemble (e)

**
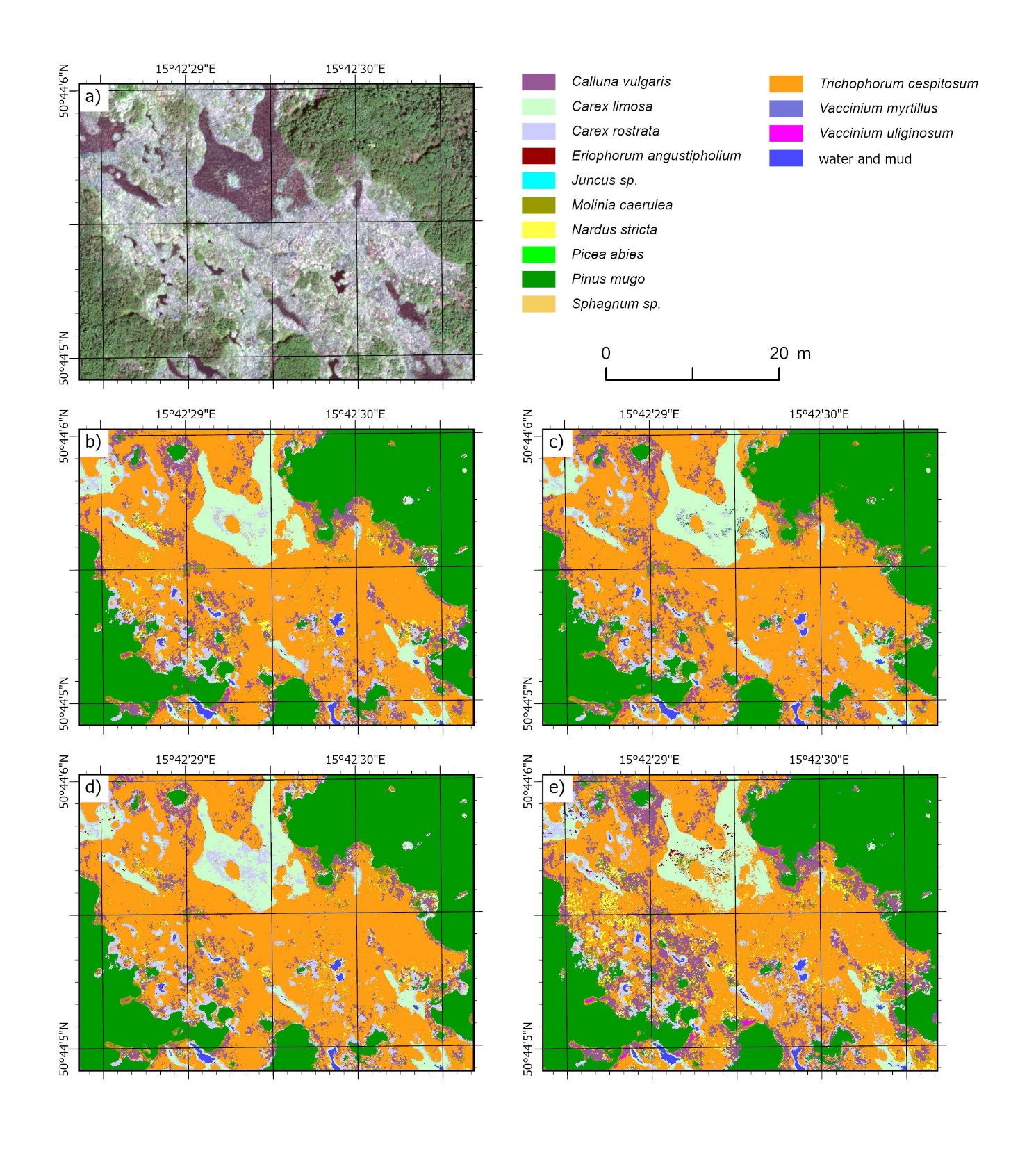
**

Figure D.4. Detailed comparison of classification results on Hraniční Meadow Peat Bog: RGB visualisation (a), XGB (b), RF (c), GBC (d), MLP ensemble (e)

**
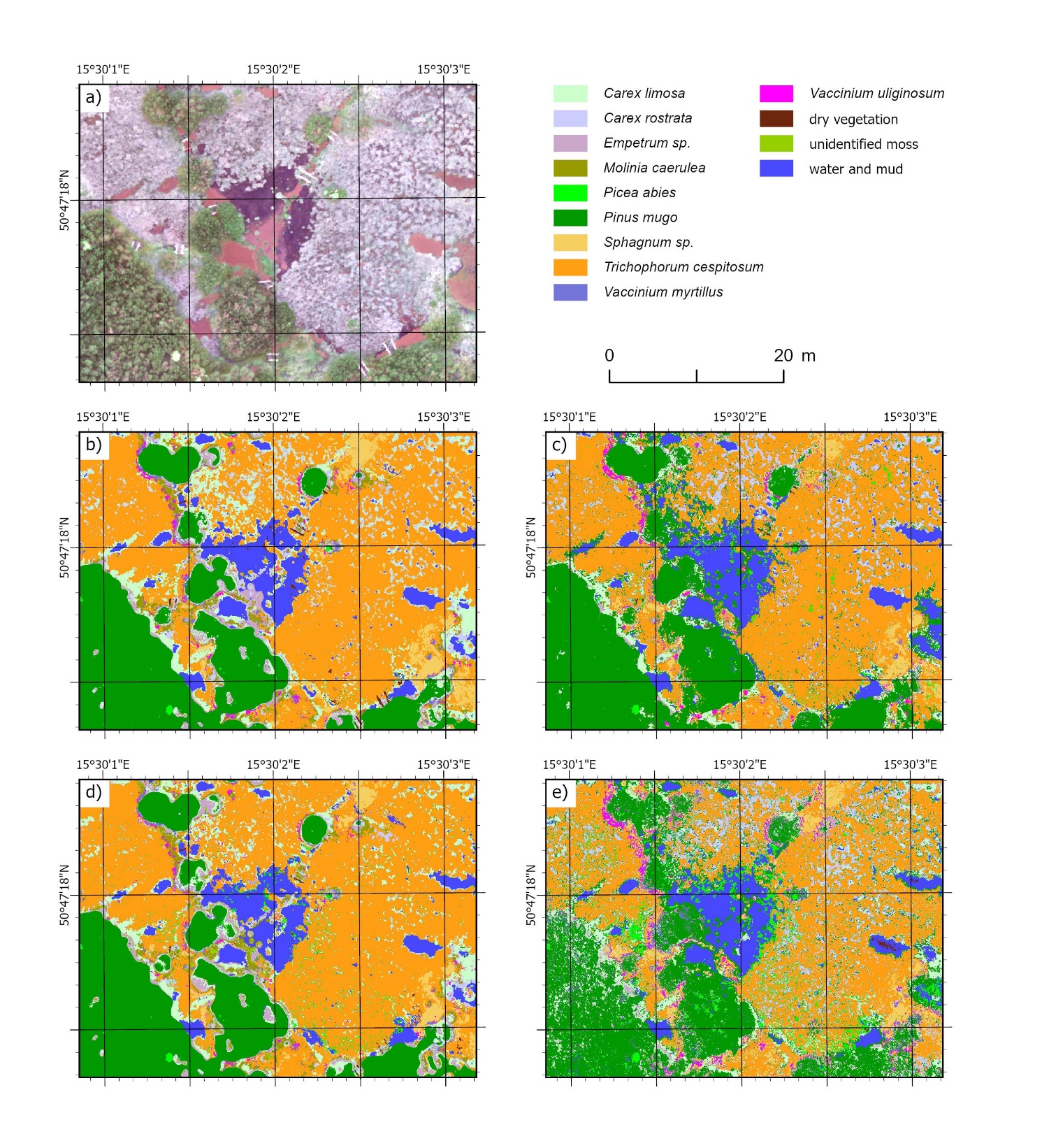
**

**Appendix E. Class separability for both sites using the same feature sets that produced the most accurate classification outputs**

Table E.1 Class separability for Úpské Peat Bog dataset (1=*Calluna vulgaris*, 2=*Carex limosa,* 3=*Carex rostrata*, 4=*Eriophorum angustifolium*, 5=*Juncus spp*., 6=*Molinia caerulea*, 7=*Nardus stricta*, 8=*Picea abies*, 9=*Pinus mugo*, 10=*Sphagnum spp.*, 11=*Trichophorum cespitosum*, 12=*Vaccinium myrtillus*, 13=*Vaccinium uliginosum*, 14=Water and mud)

|  | 1 | 2 | 3 | 4 | 5 | 6 | 7 | 8 | 9 | 10 | 11 | 12 | 13 | 14 |
| --- | --- | --- | --- | --- | --- | --- | --- | --- | --- | --- | --- | --- | --- | --- |
| 1 | 0 | 1.96 | 1.87 | 1.99 | 1.99 | 1.93 | 1.70 | 1.89 | 1.87 | 1.86 | 1.76 | 1.76 | 1.82 | 2.00 |
| 2 | 1.96 | 0 | 1.19 | 1.39 | 1.75 | 1.98 | 1.96 | 1.78 | 1.44 | 1.96 | 1.93 | 1.97 | 2.00 | 1.82 |
| 3 | 1.87 | 1.19 | 0 | 1.60 | 1.87 | 1.96 | 1.63 | 1.62 | 1.41 | 1.94 | 1.67 | 1.92 | 1.99 | 1.91 |
| 4 | 1.99 | 1.39 | 1.60 | 0 | 1.93 | 2.00 | 2.00 | 1.95 | 1.87 | 2.00 | 1.99 | 2.00 | 1.87 | 1.99 |
| 5 | 1.99 | 1.75 | 1.87 | 1.93 | 0 | 1.99 | 1.99 | 1.97 | 1.90 | 1.91 | 1.89 | 1.99 | 2.00 | 2.00 |
| 6 | 1.93 | 1.98 | 1.96 | 2.00 | 1.99 | 0 | 1.97 | 1.65 | 1.97 | 1.98 | 1.94 | 1.68 | 1.67 | 2.00 |
| 7 | 1.70 | 1.96 | 1.63 | 2.00 | 1.99 | 1.97 | 0 | 1.96 | 1.93 | 1.95 | 1.03 | 1.87 | 1.97 | 2.00 |
| 8 | 1.89 | 1.78 | 1.62 | 1.95 | 1.97 | 1.65 | 1.96 | 0 | 1.42 | 1.98 | 1.96 | 1.80 | 1.81 | 1.96 |
| 9 | 1.87 | 1.44 | 1.41 | 1.87 | 1.90 | 1.97 | 1.93 | 1.42 | 0 | 1.96 | 1.94 | 1.91 | 1.99 | 1.97 |
| 10 | 1.86 | 1.96 | 1.94 | 2.00 | 1.91 | 1.98 | 1.95 | 1.98 | 1.96 | 0 | 1.78 | 1.98 | 1.99 | 2.00 |
| 11 | 1.79 | 1.93 | 1.67 | 1.99 | 1.89 | 1.94 | 1.03 | 1.96 | 1.94 | 1.78 | 0 | 1.89 | 2.00 | 1.96 |
| 12 | 1.76 | 1.97 | 1.92 | 2.00 | 1.99 | 1.68 | 1.87 | 1.80 | 1.91 | 1.98 | 1.89 | 0 | 1.48 | 2.00 |
| 13 | 1.82 | 2.00 | 1.99 | 1.87 | 2.00 | 1.67 | 1.97 | 1.81 | 1.99 | 1.99 | 2.00 | 1.48 | 0 | 2.00 |
| 14 | 2.00 | 1.82 | 1.91 | 1.99 | 2.00 | 2.00 | 2.00 | 1.96 | 1.97 | 2.00 | 1.96 | 2.00 | 2.00 | 0 |

Table E.2 Class separability for Hraniční Meadow Peat Bog dataset (1=*Carex limosa*, 2=*Carex rostrata*, 3=*Empetrum spp*., 4=*Molinia caerulea*, 5=*Picea abies*, 6=*Pinus mugo*, 7=*Sphagnum spp.*, 8=*Trichophorum cespitosum*, 9=*Vaccinium myrtillus*, 10=*Vaccinium uliginosum*, 11=Unidentified moss, 12=Dry vegetation, 13=Water and mud)

|  | 1 | 2 | 3 | 4 | 5 | 6 | 7 | 8 | 9 | 10 | 11 | 12 | 13 |
| --- | --- | --- | --- | --- | --- | --- | --- | --- | --- | --- | --- | --- | --- |
| 1 | 0 | 1.89 | 1.89 | 1.85 | 1.97 | 1.83 | 1.86 | 1.83 | 1.96 | 1.94 | 1.98 | 2.00 | 2.00 |
| 2 | 1.89 | 0 | 1.99 | 1.97 | 1.68 | 1.59 | 1.94 | 1.87 | 1.99 | 1.99 | 1.95 | 1.99 | 2.00 |
| 3 | 1.89 | 1.99 | 0 | 1.99 | 1.99 | 1.89 | 1.98 | 1.99 | 1.92 | 1.93 | 1.99 | 2.00 | 2.00 |
| 4 | 1.85 | 1.97 | 1.99 | 0 | 1.99 | 1.97 | 1.67 | 1.72 | 1.99 | 1.93 | 1.99 | 2.00 | 2.00 |
| 5 | 1.97 | 1.68 | 1.99 | 1.99 | 0 | 1.62 | 1.99 | 1.99 | 1.99 | 2.00 | 1.99 | 1.84 | 2.00 |
| 6 | 1.83 | 1.59 | 1.89 | 1.97 | 1.62 | 0 | 1.98 | 1.96 | 1.98 | 1.99 | 1.96 | 1.99 | 2.00 |
| 7 | 1.86 | 1.94 | 1.98 | 1.67 | 1.99 | 1.98 | 0 | 1.70 | 1.91 | 1.76 | 1.97 | 2.00 | 2.00 |
| 8 | 1.83 | 1.99 | 1.99 | 1.72 | 1.99 | 1.96 | 1.70 | 0 | 1.99 | 1.95 | 1.95 | 2.00 | 2.00 |
| 9 | 1.96 | 1.99 | 1.92 | 1.99 | 1.99 | 1.98 | 1.91 | 1.99 | 0 | 1.53 | 1.99 | 2.00 | 2.00 |
| 10 | 1.94 | 1.99 | 1.93 | 1.93 | 2.00 | 1.99 | 1.76 | 1.95 | 1.53 | 0 | 1.96 | 2.00 | 2.00 |
| 11 | 1.98 | 1.95 | 1.99 | 1.99 | 1.99 | 1.96 | 1.97 | 1.95 | 1.99 | 1.96 | 0 | 2.00 | 2.00 |
| 12 | 2.00 | 1.99 | 2.00 | 2.00 | 1.84 | 1.99 | 2.00 | 2.00 | 2.00 | 2.00 | 2.00 | 0 | 2.00 |
| 13 | 2.00 | 2.00 | 2.00 | 2.00 | 2.00 | 2.00 | 2.00 | 2.00 | 2.00 | 2.00 | 2.00 | 2.00 | 0 |

# Appendix F. Feature importance values for all classes

# Table F.1 Top mean SHAP and LIME per class for Úpské Peat Bog dataset

| Class | Rank | Feature | Mean SHAP | Feature | Mean LIME |
| --- | --- | --- | --- | --- | --- |
| *Calluna vulgaris* | 1 | GLCM RedEdge mean | 1.402 | GLCM RedEdge mean | 0.0160 |
|  | 2 | CHM | 1.043 | GLCM Red mean | 0.0108 |
|  | 3 | GLCM Red mean | 0.787 | Red (July) | 0.0072 |
|  | 4 | Red (July) | 0.715 | CHM | 0.0044 |
|  | 5 | GLSZM Green homogeneity | 0.681 | GLCM CHM dissimilarity | 0.0041 |
| *Carex limosa* | 1 | GLCM RedEdge mean | 1.286 | CHM | 0.0397 |
|  | 2 | RedEdge (July) | 1.248 | GLCM RedEdge mean | 0.0266 |
|  | 3 | CHM | 1.196 | RedEdge (July) | 0.0246 |
|  | 4 | NIR (July) | 1.124 | Red (July) | 0.0129 |
|  | 5 | GLCM Green mean | 0.906 | GLCM Green mean | 0.0117 |
| *Carex rostrata* | 1 | GLCM Green mean | 1.848 | GLCM Green mean | 0.0376 |
|  | 2 | GLSZM CHM homogeneity | 1.666 | CHM | 0.0267 |
|  | 3 | GLCM NIR mean | 1.608 | Green (July) | 0.0265 |
|  | 4 | Green (August) | 1.191 | Green (August) | 0.0232 |
|  | 5 | NIR (August) | 1.173 | GLCM NIR mean | 0.0196 |
| *Eriophorum angustifolium* | 1 | CHM | 1.711 | CHM | 0.0139 |
|  | 2 | NIR (July) | 1.244 | NIR (July) | 0.0109 |
|  | 3 | NIR (August) | 1.215 | GLCM NIR mean | 0.0092 |
|  | 4 | GLCM NIR mean | 1.157 | GLCM CHM homogeneity | 0.0070 |
|  | 5 | GLSZM RedEdge homogeneity | 0.975 | GLSZM CHM homogeneity | 0.0067 |
| *Juncus spp.* | 1 | Blue (August) | 1.761 | Blue (August) | 0.0504 |
|  | 2 | Red (August) | 0.868 | CHM | 0.0244 |
|  | 3 | GLCM Green mean | 0.813 | Red (August) | 0.0115 |
|  | 4 | Green (July) | 0.471 | GLCM Green mean | 0.0088 |
|  | 5 | GLSZM CHM homogeneity | 0.454 | Red (July) | 0.0081 |
| *Molinia caerulea* | 1 | CHM | 4.173 | CHM | 0.3324 |
|  | 2 | Blue (August) | 1.290 | GLCM NIR mean | 0.0840 |
|  | 3 | GLCM NIR mean | 0.963 | Blue (August) | 0.0383 |
|  | 4 | Green (August) | 0.889 | Green (August) | 0.0315 |
|  | 5 | NIR (August) | 0.703 | NIR (August) | 0.0262 |
| *Nardus stricta* | 1 | GLCM NIR mean | 2.369 | GLCM NIR mean | 0.1080 |
|  | 2 | NIR (July) | 1.865 | NIR (July) | 0.0672 |
|  | 3 | RedEdge (July) | 1.601 | Red (August) | 0.0548 |
|  | 4 | Red (August) | 1.414 | RedEdge (July) | 0.0428 |
|  | 5 | NIR (August) | 1.208 | NIR (August) | 0.0418 |
| *Picea abies* | 1 | CHM | 1.711 | CHM | 0.0078 |
|  | 2 | GLSZM CHM homogeneity | 0.951 | GLCM Red mean | 0.0069 |
|  | 3 | Red (July) | 0.731 | GLSZM Red contrast | 0.0066 |
|  | 4 | GLSZM NIR mean | 0.662 | Green (July) | 0.0065 |
|  | 5 | NIR (July) | 0.653 | GLSZM Red mean | 0.0058 |
| *Pinus mugo* | 1 | CHM | 1.538 | CHM | 0.0103 |
|  | 2 | GLCM CHM mean | 0.491 | GLCM CHM mean | 0.0060 |
|  | 3 | Red (July) | 0.447 | GLCM CHM dissimilarity | 0.0055 |
|  | 4 | GLCM Green mean | 0.312 | GLCM CHM homogeneity | 0.0054 |
|  | 5 | Red (August) | 0.283 | GLSZM NIR homogeneity | 0.0053 |
| *Sphagnum* spp. | 1 | Red (July) | 1.749 | CHM | 0.0098 |
|  | 2 | NIR (July) | 1.719 | NIR (July) | 0.0092 |
|  | 3 | Blue (August) | 1.574 | Blue (August) | 0.0084 |
|  | 4 | GLCM Green mean | 1.429 | GLCM Green mean | 0.0068 |
|  | 5 | Red (August) | 1.405 | GLCM NIR variance | 0.0063 |
| *Trichophorum cespitosum* | 1 | Red (July) | 1.954 | CHM | 0.0647 |
|  | 2 | NIR (July) | 1.812 | Red (August) | 0.0610 |
|  | 3 | Red (August) | 1.782 | Red (July) | 0.0610 |
|  | 4 | RedEdge (August) | 0.963 | NIR (July) | 0.0397 |
|  | 5 | Green (July) | 0.815 | RedEdge (August) | 0.0315 |
| *Vaccinium myrtillus* | 1 | Red (August) | 2.092 | CHM | 0.0122 |
|  | 2 | Blue (August) | 1.618 | GLCM RedEdge mean | 0.0106 |
|  | 3 | GLCM RedEdge mean | 1.112 | Blue (August) | 0.0104 |
|  | 4 | GLSZM RedEdge homogeneity | 1.102 | Red (August) | 0.0095 |
|  | 5 | GLSZM NIR mean | 1.044 | GLSZM RedEdge homogeneity | 0.0085 |
| *Vaccinium uliginosum* | 1 | NIR (July) | 1.805 | NIR (July) | 0.0534 |
|  | 2 | GLSZM Green mean | 0.939 | CHM | 0.0401 |
|  | 3 | GLCM RedEdge mean | 0.669 | GLCM RedEdge mean | 0.0262 |
|  | 4 | Red (August) | 0.567 | GLCM Red contrast | 0.0143 |
|  | 5 | NIR (August) | 0.563 | Red (August) | 0.0138 |
| Water and mud | 1 | GLCM RedEdge mean | 2.441 | GLCM RedEdge mean | 0.0931 |
|  | 2 | RedEdge (July) | 2.303 | RedEdge (July) | 0.0883 |
|  | 3 | RedEdge (August) | 2.146 | CHM | 0.0811 |
|  | 4 | Green (July) | 1.697 | RedEdge (August) | 0.0795 |
|  | 5 | NIR (July) | 1.434 | Blue (August) | 0.0621 |

# Table F.2 Top mean SHAP and LIME per class for Hraniční Meadow Peat Bog dataset

| Class | Rank | Feature | Mean SHAP | Feature | Mean LIME |
| --- | --- | --- | --- | --- | --- |
| *Carex limosa* | 1 | Red | 0.634 | GLCM RedEdge homogeneity | 0.0018 |
|  | 2 | GLCM RedEdge homogeneity | 0.490 | Red | 0.0017 |
|  | 3 | GLCM Green homogeneity | 0.476 | GLDM RedEdge dissimilarity | 0.0015 |
|  | 4 | CHM | 0.363 | GLSZM Green entropy | 0.0014 |
|  | 5 | GLDM RedEdge homogeneity | 0.307 | CHM | 0.0014 |
| *Carex rostrata* | 1 | CHM | 1.328 | CHM | 0.0245 |
|  | 2 | GLCM NIR variance | 0.418 | GLCM Red mean | 0.0083 |
|  | 3 | GLCM Red mean | 0.269 | GLDM Red mean | 0.0059 |
|  | 4 | Red | 0.238 | Red | 0.0052 |
|  | 5 | GLDM NIR variance | 0.198 | Green | 0.0048 |
| *Empetrum* spp. | 1 | CHM | 1.555 | CHM | 0.0077 |
|  | 2 | GLCM NIR mean | 0.533 | GLCM NIR variance | 0.0038 |
|  | 3 | GLDM NIR variance | 0.463 | GLCM Red contrast | 0.0030 |
|  | 4 | GLSZM NIR maximum | 0.260 | GLCM RedEdge variance | 0.0030 |
|  | 5 | GLDM RedEdge contrast | 0.221 | GLCM NIR mean | 0.0028 |
| *Molinia caerulea* | 1 | Red | 0.843 | Red | 0.0040 |
|  | 2 | GLSZM RedEdge maximum | 0.592 | CHM | 0.0030 |
|  | 3 | CHM | 0.524 | GLSZM NIR homogeneity | 0.0023 |
|  | 4 | NIR | 0.305 | GLDM Red contrast | 0.0021 |
|  | 5 | GLSZM NIR homogeneity | 0.304 | GLCM RedEdge variance | 0.0018 |
| *Picea abies* | 1 | CHM | 3.651 | GLDM Green dissimilarity | 0.0356 |
|  | 2 | GLDM Red homogeneity | 0.278 | CHM | 0.0294 |
|  | 3 | GLDM NIR contrast | 0.275 | GLDM Red dissimilarity | 0.0243 |
|  | 4 | GLDM Green homogeneity | 0.256 | GLDM NIR contrast | 0.0237 |
|  | 5 | GLDM Red contrast | 0.207 | GLDM Red contrast | 0.0217 |
| *Pinus mugo* | 1 | CHM | 0.640 | GLSZM Red contrast | 0.0105 |
|  | 2 | GLCM RedEdge dissimilarity | 0.216 | GLDM Green variance | 0.0046 |
|  | 3 | GLCM Green dissimilarity | 0.212 | CHM | 0.0039 |
|  | 4 | Red | 0.210 | GLSZM NIR homogeneity | 0.0028 |
|  | 5 | GLDM Red homogeneity | 0.204 | GLDM RedEdge dissimilarity | 0.0025 |
| *Sphagnum* spp. | 1 | CHM | 0.827 | CHM | 0.0024 |
|  | 2 | GLDM NIR homogeneity | 0.568 | GLSZM NIR contrast | 0.0020 |
|  | 3 | GLCM Red homogeneity | 0.367 | GLDM NIR dissimilarity | 0.0020 |
|  | 4 | GLCM RedEdge variance | 0.367 | GLCM Green mean | 0.0020 |
|  | 5 | RedEdge | 0.301 | GLCM Green contrast | 0.0020 |
| *Trichophorum cespitosum* | 1 | CHM | 3.442 | CHM | 0.3798 |
|  | 2 | NIR | 0.461 | GLDM Green dissimilarity | 0.0320 |
|  | 3 | GLDM Green homogeneity | 0.261 | GLDM NIR contrast | 0.0263 |
|  | 4 | GLDM NIR contrast | 0.252 | GLDM Red contrast | 0.0230 |
|  | 5 | GLDM Red contrast | 0.206 | GLCM NIR mean | 0.0197 |
| *Vaccinium myrtillus* | 1 | CHM | 1.629 | CHM | 0.2749 |
|  | 2 | GLSZM NIR homogeneity | 0.305 | Green | 0.0136 |
|  | 3 | GLSZM NIR contrast | 0.304 | GLDM NIR variance | 0.0133 |
|  | 4 | GLCM NIR mean | 0.279 | GLSZM RedEdge mean | 0.0123 |
|  | 5 | GLSZM RedEdge mean | 0.228 | GLCM RedEdge mean | 0.0114 |
| *Vaccinium uliginosum* | 1 | NIR | 0.403 | Red | 0.0019 |
|  | 2 | GLSZM RedEdge homogeneity | 0.384 | GLDM Red dissimilarity | 0.0019 |
|  | 3 | CHM | 0.375 | GLCM NIR mean | 0.0018 |
|  | 4 | GLDM Red homogeneity | 0.356 | NIR | 0.0016 |
|  | 5 | GLCM NIR mean | 0.294 | GLSZM NIR homogeneity | 0.0015 |
| Unidentified moss | 1 | CHM | 1.507 | CHM | 0.0170 |
|  | 2 | Red | 0.532 | GLSZM Red contrast | 0.0087 |
|  | 3 | GLSZM Green mean | 0.350 | Red | 0.0080 |
|  | 4 | GLSZM Red homogeneity | 0.275 | GLDM NIR variance | 0.0064 |
|  | 5 | GLDM RedEdge homogeneity | 0.244 | GLDM Green dissimilarity | 0.0041 |
| Dry vegetation | 1 | Red | 0.531 | RedEdge | 0.0016 |
|  | 2 | RedEdge | 0.471 | NIR | 0.0016 |
|  | 3 | CHM | 0.430 | CHM | 0.0015 |
|  | 4 | GLSZM NIR contrast | 0.418 | GLCM NIR maximum | 0.0015 |
|  | 5 | GLSZM RedEdge maximum | 0.357 | Red | 0.0014 |
| Water and mud | 1 | CHM | 0.976 | CHM | 0.0221 |
|  | 2 | GLCM RedEdge mean | 0.514 | GLDM NIR variance | 0.0169 |
|  | 3 | GLDM NIR variance | 0.409 | GLCM RedEdge mean | 0.0104 |
|  | 4 | NIR | 0.279 | GLSZM NIR mean | 0.0072 |
|  | 5 | GLSZM RedEdge homogeneity | 0.133 | NIR | 0.0061 |
